# Neuropeptide Precursor VGF Promotes Liver Metastatic Colonization of Gαq Mutant Uveal Melanoma by Facilitating Tumor Microenvironment via Paracrine Loops

**DOI:** 10.1002/advs.202407967

**Published:** 2024-10-18

**Authors:** Shumin Ouyang, Shuo Shi, Wen Ding, Yang Ge, Yingxue Su, Jianshan Mo, Keren Peng, Qiyi Zhang, Guopin Liu, Wei Xiao, Peibin Yue, Jinjian Lu, Yandong Wang, Xiaofeng Xiong, Xiaolei Zhang

**Affiliations:** ^1^ National‐Local Joint Engineering Laboratory of Druggability and New Drug Evaluation Guangdong Key Laboratory of Chiral Molecule and Drug Discovery School of Pharmaceutical Sciences Sun Yat‐sen University Guangzhou 510006 China; ^2^ State Key Laboratory of Ophthalmology Zhongshan Ophthalmic Center Sun Yat‐sen University Guangzhou 510060 China; ^3^ Department of Medicine Division of Hematology‐Oncology and Samuel Oschin Comprehensive Cancer Institute Cedars‐Sinai Medical Center Los Angeles CA 90048 USA; ^4^ State Key Laboratory of Quality Research in Chinese Medicine Institute of Chinese Medical Sciences University of Macau Macao 999078 China

**Keywords:** Gαq mutant, hepatic stellate cells, liver metastasis, tumor microenvironment, uveal melanoma, VGF

## Abstract

Uveal melanoma (UM), the predominant primary ocular malignancy, often progresses to liver metastasis with limited therapeutic options. The interplay of the tumor microenvironment, encompassing secreted soluble factors, plays a crucial role in facilitating liver metastasis. In this study, the role is elucidated of the neural growth factor‐inducible gene (VGF), a secreted neuropeptide precursor, in Gαq mutant UM. Employing a multiomics approach, encompassing transcriptomic and secretomic analyses, the intricate involvement of VGF in UM progression is unveiled. VGF is upregulated in Gαq mutant UM cells and associated with poor prognosis of UM patients. Targeting VGF significantly suppressed the growth of UM in vitro and in vivo. Further evidence shows that VGF is regulated by Gαq through MAPK/CREB pathway. Mechanistically, CREB modulates VGF expression by directly binding to consensus DNA response elements in the promoters of the VGF gene. Combined inhibition of Gαq and MEK remarkably reduces tumor burden in the UM xenograft model. Notably, VGF triggers liver metastatic colonization of UM and activates the fibrosis of hepatic stellate cells (HSCs), creating a favorable microenvironment, through an autocrine and paracrine loop. Furthermore, VGF directly binds to TGFBR2 and regulates TGF‐β‐SMAD signaling pathway, thereby regulating genes associated with endothelial‐mesenchymal transition (EMT) to promote metastasis. Taken together, these findings identify VGF as a pivotal driver in the progression and metastasis of Gαq mutant UM and confers a promising therapeutic target and strategy for UM patients.

## Graphical abstract

The secreted neuropeptide precursor, VGF, has been identified as a facilitator of the progression and metastasis of Gαq mutant Uveal melanoma (UM) through intricate autocrine and paracrine signaling loops. Moreover, its regulation by Gαq is mediated through the MAPK‐CERB axis, and VGF has the capability to bind to TGFBR2, thereby modulating the TGF‐β‐SMAD signaling pathway, and activating hepatic stellate cells. Consequently, these findings underscore VGF as a promising therapeutic target and biomarker for metastatic UM.

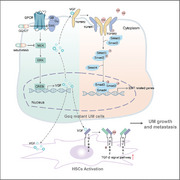

## 1 Introduction

Uveal melanoma (UM) is the most common primary intraocular malignancy in adults,^[^ ^1^ ^]^ and mainly occurs in the choroid (90%), ciliary body (6%), and iris (4%). UM patients are usually at advanced stages when diagnosed, due to insidious onset and ignorable early symptoms. More than that, almost 50% of patients with UM develop metastatic disease,^[^ ^2^ ^]^ and is usually fatal within 1 year. Liver is the most common site of metastases,^[^ ^1^ ^]^ and the one‐year and two‐year overall survival rates of patients with liver metastases are 13% and 8%, respectively.^[^ ^3^ ^]^ Although treatment of the primary tumor is effective, UM metastases respond poorly to chemotherapy and targeted therapy and generally do not respond to immune checkpoint inhibitors. Currently, effective therapies to prevent the development of metastases are not available. Hence, there is an urgent need to explore the underlying mechanisms and develop new and effective therapeutic approaches to prevent and treat the deadly UM metastases.

More than 85% of UMs are initiated by a mutation in G protein subunit alpha q (GNAQ) or alpha 11 (GNA11),^[^ ^4^ ^]^ among which 95% are a Q209P/L single‐site mutation,^[^ ^5^ ^]^ which is a pivotal carcinogenic factor in the pathogenesis and progression of UM. GNAQ and GNA11 encode α subunits (αq family) of the heterotrimeric G proteins, which usually activate multiple intracellular pathways in response to activation, including mitogen‐activated protein kinase (MAPK),^[^ ^6^ ^]^ RhoA‐Rac and Yes‐associated protein (YAP).^[^ ^7^ ^]^ Therefore, the mutant Gαq protein is a promising therapeutic target for the discovery of novel compounds against UM. Unfortunately, drugs that directly inhibit Gαq, such as YM‐254890^[^ ^8^ ^]^ and FR900359,^[^ ^9^ ^]^ do not distinguish between wild‐type and mutated Gαq, and are difficult to be isolated from natural sources and synthesized, making them toxic and unsuitable for clinical application. Furthermore, current inhibitors of G protein pathway downstream effectors (such as MEK and PKC) show limited clinical benefit in UM treatment.^[^ ^10^, ^11^ ^]^ Thus, it is urgent to explore new targets and mechanism of driving the progression and metastasis of Gαq mutated UM to improve UM therapy.

Tumor‐secreted proteins are major mediators for cancer cells to adapt and modify foreign microenvironments, which play an important role in cancer progression and metastasis.^[^ ^12^ ^]^ The neurosecretory protein VGF, belongs to the granin family of neuropeptides, distinctively composed of acidic secretory proteins.^[^ ^13^ ^]^ VGF contains ≈12 cleavage sites and can be cleaved into different peptides with specific neuronal biological activity.^[^ ^14^ ^]^ VGF and VGF‐derived peptides are essential for energy balance regulation, neurogenesis, synaptogenesis, learning and memory, and depression‐related behaviors.^[^ ^15^ ^]^ VGF is also associated with many neurodegenerative and psychiatric diseases,^[^ ^16^ ^]^ such as Alzheimer's disease, Huntington's disease, Parkinson's disease, amyotrophic lateral sclerosis, dementia with lewy bodies, and mood disorders. To date, studies of VGF have mainly focused on its functions in neurogenesis and synaptogenesis. A few studies also reported the role of VGF in cancer progression, such as TKI resistance in lung cancer,^[^ ^17^ ^]^ and survival and stemness of glioblastoma.^[^ ^18^ ^]^ However, the functional role of VGF in UM remains unclear.

Here, we show that VGF, a secreted neuropeptide precursor, promotes growth and liver metastasis of Gαq mutant UM through TGF‐β/SMAD signaling pathway, and activates HSCs. Expression and secretion of VGF was upregulated in Gαq‐mutant UM cells. High VGF expression is correlated with reduced overall survival and disease‐free survival in UM patients. Knockdown of VGF significantly dampens the growth ability of Gαq‐mutant UM in vitro and in vivo. VGF is regulated by Gαq through MAPK/CREB pathway, where CREB binds to consensus DNA response elements in the promoters of VGF. Combined inhibition of Gαq and MEK significantly reduced tumor burden in the UM xenograft model. Moreover, VGF promotes cell migration, invasion and spheroid formation in UM cells, and enhances liver metastatic colonization of UM in vivo, which is alleviated by our Gαq inhibitor GQ127. Furthermore, VGF regulates TGF‐β‐SMAD signaling by directly binding to TGFBR2 to promote the progression and metastasis of UM. Additionally, VGF triggers the activation of HSCs, and the activated HSCs further make feedback to enhance the ability of Gαq mutant UM cell invasion, thus facilitating liver metastatic colonization of UM. Collectively, this study identifies VGF as a key driver for Gαq mutant UM progression and metastasis, and provides a biomarker and a new therapeutic strategy for metastatic UM.

## 2 Results

### 2.1 VGF Secretion and Expression are Associated with Gαq Mutant Uveal Melanoma

**Figure 1:**
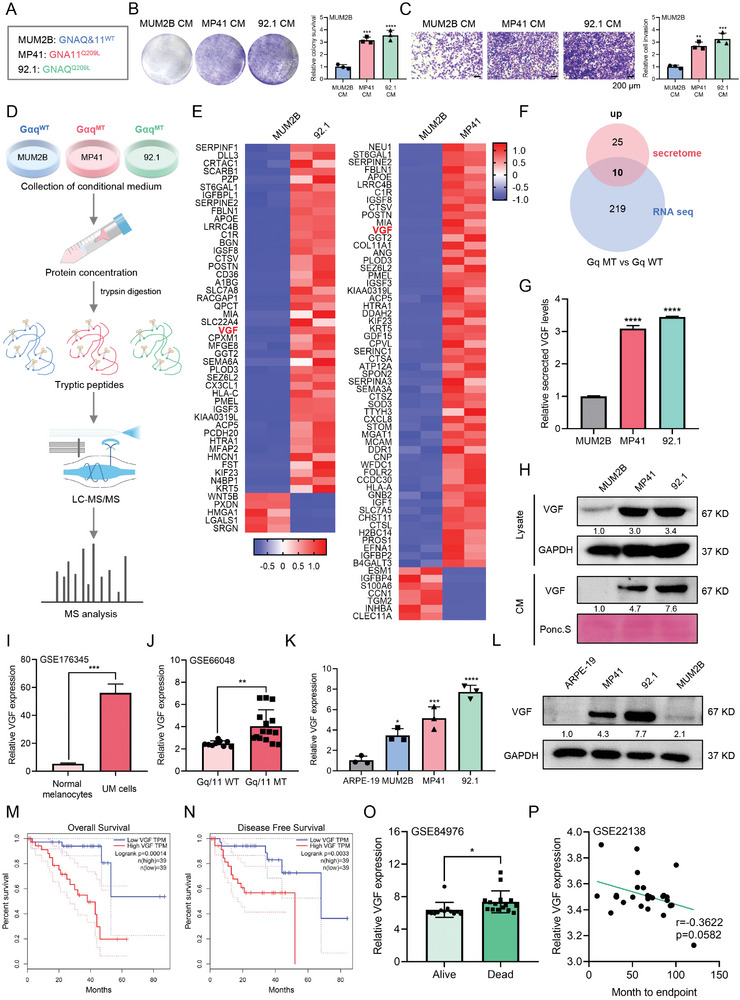
VGF secretion and expression are associated with Gαq mutant UM. A) Three UM cell lines with different types of Gαq were applied to perform experiments. GNAQ^WT^ and GNA11^WT^ UM cell line MUM2B, GNA11^Q209L^ UM cell line MP41, and GNAQ^Q209L^ UM cell line 92.1. B) Colony survival assay in MUM2B cells after cultured with MUM2B, MP41, or 92.1 CM for 7 d. Right: quantification of colony number (Fold change). CM, conditional medium. Data were presented as mean ± SD, *n*  =  3. ***p < 0.001, ****p < 0.0001 by using the one‐way ANOVA. C) Cell invasion assay in MUM2B cells after cultured with MUM2B, MP41 or 92.1 CM for 24 h. Scale bars = 200 µm. Right: quantification of cell invasion (Fold change). Data were presented as mean ± SD, *n*  =  3. **p < 0.01, ***p < 0.001 by using the one‐way ANOVA. D) Schematics of the secretome analysis (*n* = 2 per group). E) Heatmap of MS secretomic analyses of UM cell lines with different types of Gαq in (D). F) Venn diagrams showed overlapped potential targets for Gαq mutant UM cells by secretome and RNA‐seq (GEO: GSE33655 and GSE66048). Fold change [FC] > 2, *p* < 0.05. G) VGF secretion was measured by ELISA assay. Data were presented as mean ± SD, *n*  =  3. ****p < 0.0001 by using the one‐way ANOVA. H) Western blotting analysis of UM cell lines (lysates or CM) with different types of Gαq. I) The expression of VGF in UM cells compared with normal melanocytes in GSE176345. Data were presented as mean ± SD, *n*  =  3. ***p < 0.001 by using two‐tailed unpaired Student *t*‐test. J) The expression of VGF in Gαq mutant UM cells compared with Gαq wild‐type UM cells in GSE197656. Data were presented as mean ± SD, Gq/11 WT group (*n* = 9), Gq/11 MT group (*n* = 15). **p < 0.01 by using two‐tailed unpaired Student *t*‐test. K,L) The mRNA (K) and protein (L) expression of VGF in normal human retinal pigment epithelial ARPE‐19 cells and UM cells. Data in (K) were presented as mean ± SD, *n*  =  3. *p < 0.05, ***p < 0.001, ****p < 0.0001 by using the one‐way ANOVA. M,N) The overall survival analysis (M) and the disease‐free survival analysis (N) of UM patients from TCGA project with VGF high or low expression levels (defined by RNA sequencing with group cutoff in high‐50% (*n* = 39)/low‐50% (*n* = 39)) in UVM‐TCGA database. O) The expression of VGF in alive and dead UM patients in GSE84976. Data were presented as mean ± SD, alive group (*n* = 12), dead group (*n* = 16). *p < 0.05 by using two‐tailed unpaired Student *t*‐test. P) The correlation analysis between VGF expression and month to endpoint of UM patients in GSE22138. *r* and *p* value were measured using Pearson's correlation test.

We recently compared the proliferating effects and prometastatic potential of conditioned medium (CM) obtained from wild‐type or mutated Gαq UM cells. Three different UM cell lines were applied to perform experiments, including GNAQ^WT^ and GNA11^WT^ UM cell line MUM2B, GNA11^Q209L^ UM cell line MP41, and GNAQ^Q209L^ UM cell line 92.1(**Figure** **1**A). Interestingly, we found that MUM2B cells showed a stronger ability for proliferation, survival and invasion when cultured in CM obtained from GNAQ/11 mutant UM cells (MP41 and 92.1) (Figure 1B,C; Figure S1A, Supporting Information). We speculated that certain tumor‐secreted soluble factors that exist in CM obtained from mutated Gαq UM cells might have pro‐growth and metastasis potential. To investigate tumor‐secreted proteins associated with Gαq mutant UM, we further performed mass spectrometric (MS) secretomic profiling analysis of these UM cell line series with varied types of Gαq (Figure 1D,E). Based on the intersection of differentially expressed genes (DEGs) in the two sets of comparisons (MP41 CM vs MUM2B CM, and 92.1 CM versus MUM2B CM), 35 upregulated proteins overlapped and were associated with Gαq mutant UM (Figure 1E; Figure S1B, Supporting Information). We further compared DEGs from the transcriptome analysis of Gαq mutant and wild‐type UM cells in the databases of two independent clinical GEO cohorts (GSE33655 and GSE66048) (Figure S1C,D, Supporting Information). Overlapping analysis of the secretome data and RNA seq data identified 10 extracellular proteins that were consistently upregulated in Gαq mutant UM (Figure 1F; Figure S1E, Supporting Information).

VGF was among the upregulated proteins, and further analyses confirmed that its expression and secretion were elevated in Gαq mutant UM cells (Figure 1G,H). It was also found that VGF was significantly upregulated in UM cells as compared with normal melanocytes (GSE176345 and GSE181125) (Figure 1I; Figure S1F, Supporting Information). Consistently, VGF was highly expressed in UM tissue compared to adjacent tissues (Figure S1G, Supporting Information). The upregulation of VGF in Gαq mutant UM was also observed in three independent GEO data (Figure 1J; Figure S1H,I, Supporting Information). Furthermore, we detected enhanced mRNA and protein expression of VGF in three UM cell lines, especially Gq mutant UM cells, compared with that in normal human retinal pigment epithelial ARPE‐19 cells (Figure 1K,L, Supporting Information). To determine the clinical relevance of VGF in human UM, we analyzed several cohorts of human UM tumors based on the TCGA and GEO database (GSE84976 and GSE22138), and found that higher VGF expression was correlated with the poor prognosis of patients (Figure 1M–P, Supporting Information). Overall, these data demonstrated a potential oncogenic role of VGF in Gαq mutant UM.

### 2.2 VGF Promotes Gαq Mutant Uveal Melanoma Cell Growth In Vitro and In Vivo

**Figure 2:**
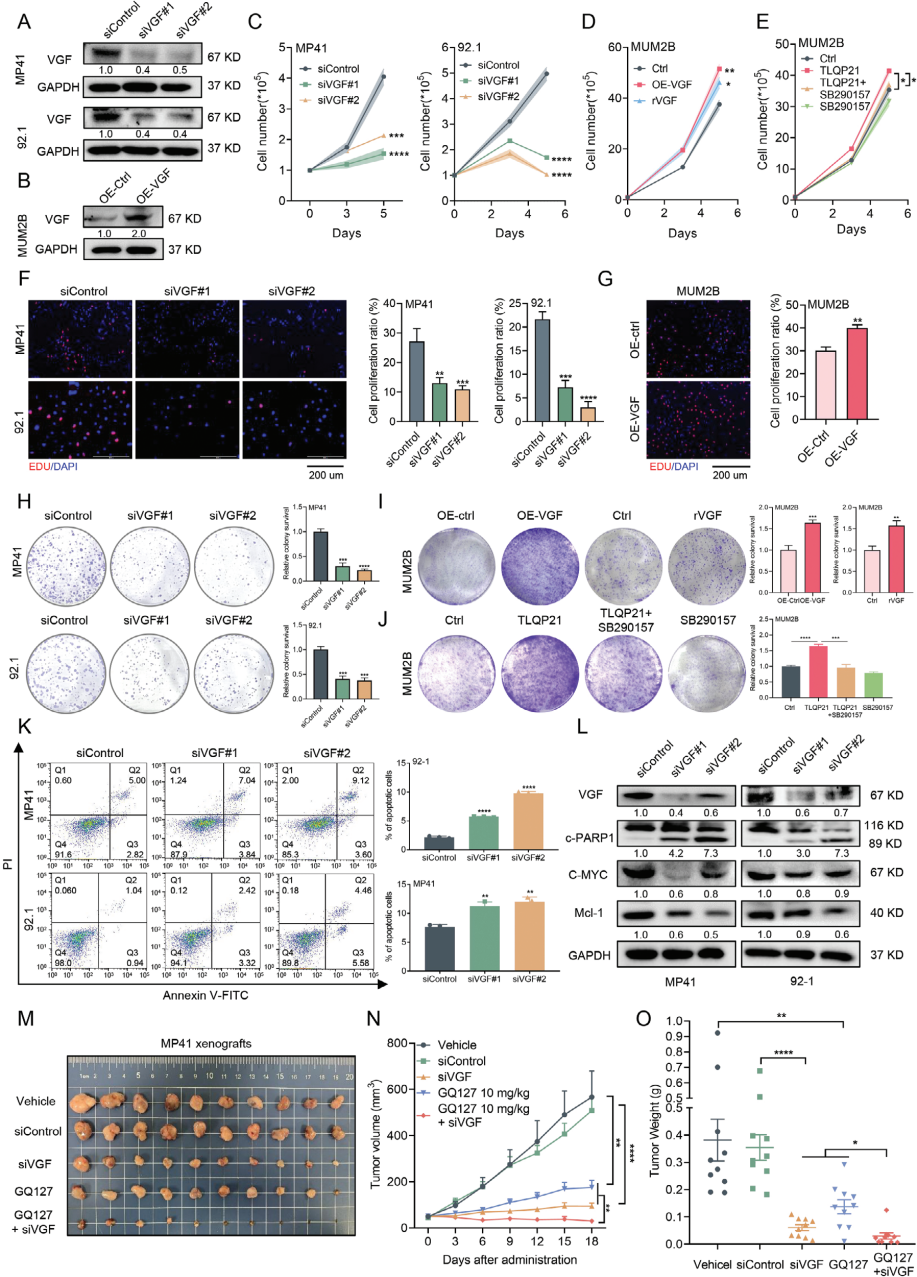
VGF promotes Gαq mutant UM cell proliferation in vitro and in vivo. A) The knockdown effect of VGF by siRNAs in MP41 and 92.1 cells was determined by immunoblotting analysis. B) The overexpression of VGF level in MUM2B cells was determined by immunoblotting analysis. C) Cell growth determined by cell numbers in UM cells (MP41 and 92.1) treated with siRNAs against VGF or control siRNA (siControl). Viable cells were counted at indicated time points (day3 and day5). Data were presented as mean ± SEM, *n*  =  3. ***p < 0.001, ****p < 0.0001 by using the one‐way ANOVA. D) Cell growth determined by cell numbers in MUM2B cells treated with control, VGF overexpression or rVGF (100 ng mL^−1^). rVGF, recombinant human VGF protein. Viable cells were counted at indicated time points (day3 and day5). Data were presented as mean ± SEM, *n*  =  3. *p < 0.05; **p < 0.01 by using the one‐way ANOVA. E) Cell growth determined by cell numbers in MUM2B cells treated with control, TLQP21 (10 × 10^−6^ m) or SB290157 (4 × 10^−6^ m). Viable cells were counted at indicated time points (day3 and day5). Data were presented as mean ± SD, *n* = 3. *p < 0.05 by using two‐tailed unpaired Student *t*‐test. F) Cell proliferation determined by EDU staining in MP41 and 92.1 cells treated with siRNAs against VGF or control siRNA for 72 h. Scale bars = 200 µm. Right: quantification of cell proliferation. Data were presented as mean ± SD, *n* = 3. **p < 0.01, ***p < 0.001, ****p < 0.0001 by using two‐tailed unpaired Student *t*‐test. G) Cell proliferation determined by EDU staining in MUM2B cells treated with control or VGF overexpression for 72 h. Scale bars = 200 µm. Right: quantification of cell proliferation. Data were presented as mean ± SD, *n* = 3. **p < 0.01 by using two‐tailed unpaired Student *t*‐test. H) MP41 or 92.1 cells (2 × 10^3^ cells) were seeded into 6‐well plates treated with siRNAs against VGF or control siRNA for 14 d. Right: quantification of colony number (Fold change). Data were presented as mean ± SD, *n* = 3. ***p < 0.001, ****p < 0.0001 by using two‐tailed unpaired Student *t*‐test. I) MUM2B cells (1 × 10^3^ cells) were seeded into 6‐well plates treated with control, VGF overexpression or rVGF (100 ng mL^−1^) for 10 d. Left: VGF overexpression; Right: rVGF treatment. Data were presented as mean ± SD, *n* = 3. **p < 0.01, ***p < 0.001 by using two‐tailed unpaired Student *t*‐test. J) Cell survival determined by colony formation in MUM2B cells (1 × 10^3^ cells) treated with control, TLQP21(10 × 10^−6^ m) or SB290157(4 × 10^−6^ m) for 12 d. Right: quantification of colony number (Fold change). Data were presented as mean ± SD, *n* = 3. ***p < 0.001, ****p < 0.0001 by using two‐tailed unpaired Student *t*‐test. K) Cell apoptosis detected by flow cytometry in MP41 and 92.1 cells treated with siRNAs against VGF or control siRNA for 72 h. Right: quantification of cell apoptosis. Data were presented as mean ± SD, *n* = 3. **p < 0.01, ****p < 0.0001 by using two‐tailed unpaired Student *t*‐test. L) Western blotting analysis of the indicated proteins in MP41 and 92.1 cells treated with siRNAs against VGF or control siRNA for 72 h. M–O) BALB/c‐nu/nu mice bearing the MP41 subcutaneous xenografts received the vehicle, GQ127 (intraperitoneal injection, 10 mg kg^−1^), once daily; siVGF or siControl (intratumoral injection) every 3 d (*n* = 10 per group). Representative tumor images (M), Mean tumor volume ± standard error of the mean (SEM) (N), and mean tumor weight ± SEM (O)are shown. Data were presented as mean ± SEM, *n* = 10. *p < 0.05, **p < 0.01, ****p < 0.0001 by using two‐tailed unpaired Student *t*‐test.

To explore the functional role of VGF in UM, we knocked down VGF by small interfering RNAs in Gαq mutant UM cells and forced expression of VGF in Gαq wild‐type UM cells. The mRNA and protein expression of VGF were verified by qRT‐PCR and immunoblotting in UM cells (**Figure** **2**A,B; Figure S2A‐B, Supporting Information). We found that the knockdown of VGF significantly inhibited the growth of both 92‐1 and MP41 cells with Gαq mutation (Figure 2C), whereas its impact on MUM2B cells that harbor wild‐type Gαq or non‐tumor cells (ARPE‐19 and HLE‐B3) was comparatively limited (Figure S2C–E, Supporting Information). In contrast, VGF overexpression significantly exacerbated cell growth in UM cells (Figure 2D). Importantly, recombinant human VGF protein (rVGF) treatment also effectively promoted cell growth in UM cells (Figure 2D). Given that TLQP21 (VGF_554‐574_)^[^ ^19^ ^]^ is an extensively studied VGF‐derived peptide that has been proven to have a definite biological function,^[^ ^20^ ^]^ we also investigated the proliferation potential of TLQP‐21 in UM cells. In addition, TLQP21 has been shown to signal via gC1qR^[^ ^21^ ^]^ and C3aR1^[^ ^22^ ^]^ receptors, which are Gq coupled to GPCR. Furthermore, we observed that TLQP‐21 treatment also promoted cell growth in UM cells, whereas C3aR inhibitor SB290157 attenuated the effect of TLQP‐21(Figure 2E). Concordantly, our data showed that VGF knockdown significantly reduced cell proliferation (Figure 2F) and survival (Figure 2H) with EDU staining and colony formation assay, while overexpression of VGF (Figure 2G,I), rVGF (Figure 2I) and TLQP21(Figure 2J) treatment promoted UM cell survival. Moreover, our flow cytometry and immunoblotting results both showed that VGF knockdown resulted in a significant increase in cell apoptosis (Figure 2K,L). The expression of cleaved PARP‐1 (apoptosis‐related gene) was upregulated, while the expression of C‐MYC and Mcl‐1 (proliferation‐related genes) was downregulated in UM cells (Figure 2L).

Next, we established subcutaneous mouse xenografts of MP41 cells to further evaluate the effect of VGF in vivo. Consistently, the knockdown of VGF remarkably inhibited the growth and tumor weight of UM xenografts (Figure 2M–O). And the novel Gq inhibitor GQ127,^[^ ^23^ ^]^ developed by our lab previously, also showed a strong antitumor effect (Figure 2M–O). Intriguingly, the combination of siVGF and GQ127 eradicates tumor cells in UM xenografts, by extremely decreased tumor size and weight (Figure 2M–O). Immunohistochemical staining of the tumor sections demonstrated that VGF knockdown and Gαq inhibition decreased the expression of VGF and Ki67 (cell proliferation) (Figure S2F, Supporting Information). Moreover, there were no significant changes in body weight or obvious signs of toxicity during the treatment (Figure S2G, Supporting Information). Histological analysis of the tissues from the lung, heart, liver, kidney, and spleen, further confirmed that the treatment had no obvious toxicity (Figure S2H,I, Supporting Information). These findings demonstrated a proliferating role of VGF in vitro and in vivo, implying that VGF is a potential therapeutic target in Gαq mutant UM.

### 2.3 VGF is Regulated by Gαq Through the MAPK/CREB Pathway in Gαq Mutant Uveal Melanoma

**Figure 3:**
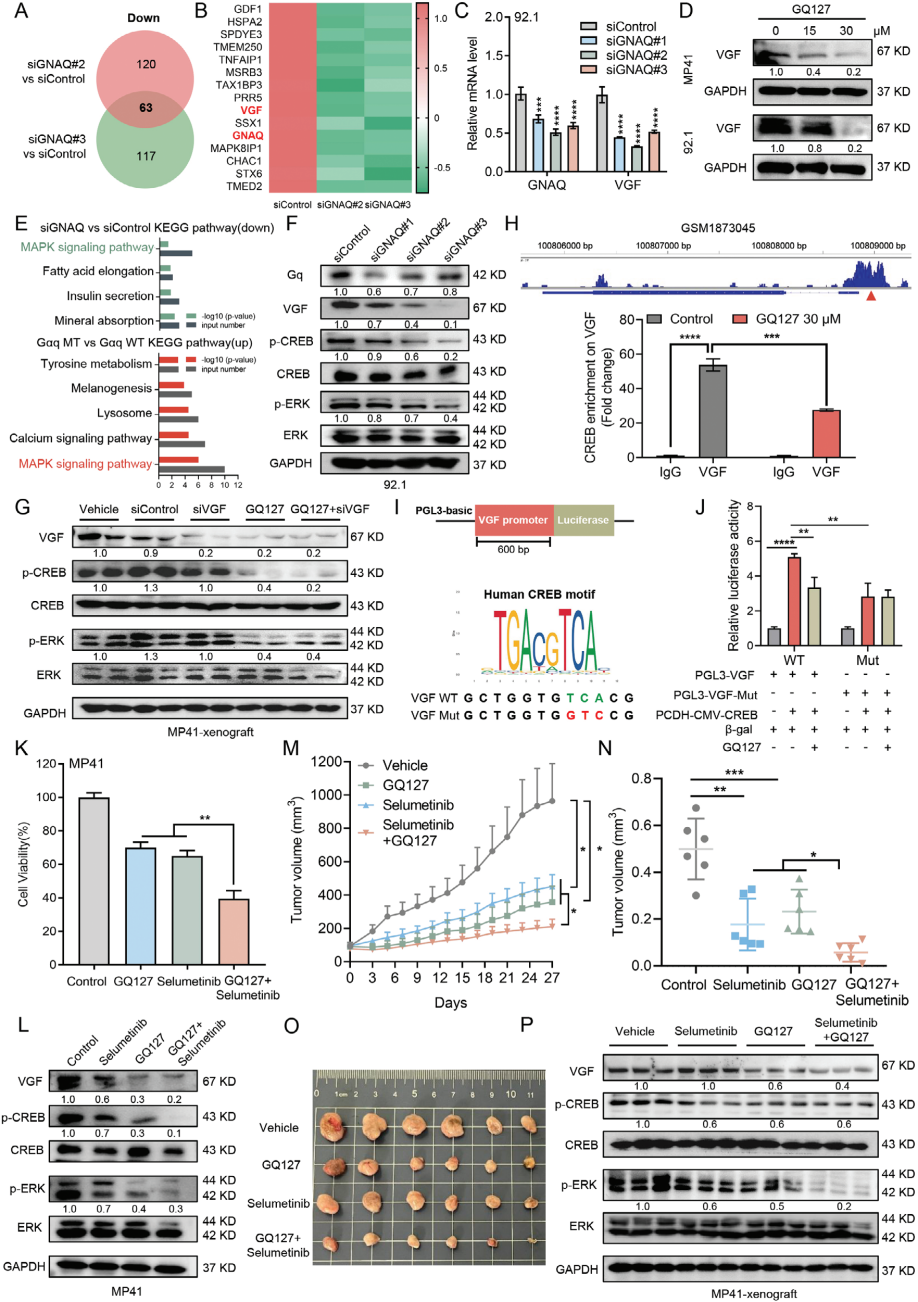
VGF is regulated by Gαq through MAPK/CREB pathway in Gαq mutant uveal melanoma. A) The overlapping of GNAQ‐downregulated mRNAs of two independent siRNA in UM cells detected by RNA‐seq. Fold change [FC] > 2, *p* < 0.05. B) The representative genes of downregulated in RNA‐seq data. C) Validation of the mRNA expression of GNAQ and VGF in siControl and siGNAQ (#1, #2, and #3) in 92.1 cells by qRT‐PCR. Data were presented as mean ± SD, *n*  =  3. ***p < 0.001, ****p < 0.0001 by using the one‐way ANOVA. D) The protein expression of VGF in UM cells treated with GQ127 (15 and 30 × 10^−6^ m) for 24 h. E) Down‐regulated pathway of GNAQ knockdown and up‐regulated pathway in Gαq mutant cells detected by RNA seq. Top: our RNA seq data; bottom: GSE33655. F) Immunoblotting of the indicated proteins in 92.1 cells treated with siControl and siGNAQ (#1, #2, and #3) for 72 h. G) Immunoblotting of the indicated proteins in MP41 xenografts. H) The previous ChIP‐seq (GSM1873045) shows that CREB has a significant binding peak in the VGF promoter region (top). ChIP‐qPCR analysis of relative enrichment of VGF at CREB gene in MP41 cells treated with vehicle or GQ127 (30 × 10^−6^ m) for 48 h (bottom). Data were presented as mean ± SD, *n* = 3. ***P < 0.001, ****P < 0.0001 by using two‐tailed unpaired Student *t*‐test. I) Promoter of VGF containing human CREB motif was cloned into luciferase reporter vector (top). Sequence of the wild‐type and mutant VGF promoter cloned to the reporter (bottom). J) VGF (wild type or mutated) promoter‐luciferase reporter activity by CREB overexpression or treatment with GQ127 (30 × 10^−6^ m) in HEK‐293T cells. Data were presented as mean ± SD, *n* = 3. **p < 0.01, ****p < 0.0001 by using two‐tailed unpaired Student *t*‐test. K) Cell growth determined by cell numbers in MP41 cells treated with GQ127 (15 × 10^−6^ m) or selumetinib (1 × 10^−6^ m) for 72 h. Data were presented as mean ± SD, *n* = 3. **p < 0.01 by using two‐tailed unpaired Student *t*‐test. L) Immunoblotting of the indicated proteins in MP41 cells treated with GQ127(15 × 10^−6^ m) or selumetinib (1 × 10^−6^ m) for 48 h. M–O) BALB/c‐nu/nu mice bearing MP41 xenografts received GQ127 (10 mg kg^−1^, i.p, daily), Selumetinib (20 mg kg^−1^, oral, daily), or vehicle daily (*n* = 6 per group). Mean tumor volume ± SEM (M), mean tumor weight (N), and representative tumor images (O) are shown. Data were presented as mean ± SEM, *n* = 6. *p < 0.05, **p < 0.01, ***p < 0.001 by using two‐tailed unpaired Student *t*‐test. P) Immunoblotting analysis of MP41 xenografts tumor tissues as in (O).

Given that the expression and secretion of VGF is upregulated in Gαq mutant UM compared with Gαq wild‐type UM, we next investigated the molecular mechanism of how Gαq regulates VGF. We performed transcriptome sequencing analysis of 92.1 cells with GNAQ knockdown. Overlapping analysis identified 63 genes that consistently downregulated in Gαq mutant UM cells (**Figure** **3**A), including GNAQ and VGF (Figure 3B). Subsequent qRT‐PCR analysis confirmed that VGF was significantly down‐regulated in response to GNAQ depletion (Figure 3C). Besides, our novel Gαq inhibitor GQ127 also inhibited the protein expression of VGF in a dose‐dependent manner (Figure 3D).

To further identify the potential signaling pathway of Gαq regulating VGF, we comprehensively analyzed the down‐regulated pathway after Gαq inhibition by siRNA (our dataset) and the up‐regulated pathway in Gαq mutant UM cells (GSE33655) (Figure 3E). Then, the above two cohorts are overlapped, and the MAPK signal pathway is further determined (Figure 3E), which is reported to be triggered by G protein mutation and an important mechanism driving the occurrence and development of UM.^[^ ^4^, ^24^ ^]^ The cAMP response element binding protein (CREB) is a cellular transcription factor dependent on the MAPK signaling pathway, which was reported to be involved in the regulation of VGF.^[^ ^25^ ^]^ We hypothesize that MAPK/CREB signaling pathways might be related to the regulation of VGF by Gαq in UM. Then, we confirmed that the protein levels of VGF and the activation of CREB and ERK were remarkably decreased in GNAQ‐knockdown UM cells (Figure 3F). Consistent with in vitro experiments, immunoblotting analysis of MP41 xenograft tumors demonstrated that VGF expression was significantly decreased in GQ127 or siVGF alone and the combinational treatment groups (Figure 3G). Downregulation of p‐ERK and p‐CREB was also observed in GQ127 alone and the combination group (Figure 3G).

As previous studies have suggested that the classical cellular transcription factor CREB may regulate the expression of VGF,^[^ ^26^ ^]^ we questioned whether CREB regulates VGF in a transcriptional manner in Gαq mutant UM cells. The ChIP‐seq of CREB in previous studies identified VGF as a potential target gene of CREB.^[^ ^26^ ^]^ To directly investigate the regulation of CREB on VGF, we performed ChIP‐qPCR analysis and found that the promoter of VGF occupied by CREB in MP41 cells, and Gαq inhibitor GQ127 can partly reduce this binding (Figure 3H). To further prove the effect of CREB regulating VGF expression, we cloned the promoter region of VGF into a luciferase reporter construct and generated one mutant by replacing TCA with GTC in the core motif of the putative human CREB binding site (Figure 3I). The relative luciferase activity of the VGF promoter was enhanced by forced expression of CREB, whereas the mutation of the VGF promoter effectively diminished CREB‐induced luciferase expression (Figure 3J). Notably, GQ127 significantly inhibited CREB‐induced VGF‐dependent luciferase activity (Figure 3J). Overall, these results demonstrated that mutant Gαq protein promotes VGF expression and secretion by activating the MAPK/CREB pathway in UM.

Given that constant activity of Gαq causes deregulation of MAPK signaling pathways, several clinical trials of MEK inhibitors against UM have been conducted in recent years. However, selumetinib, an orally available MEK1/2 inhibitor,^[^ ^27^, ^28^ ^]^ has shown limited clinical efficacy as monotherapy in metastatic UM in phase II and III clinical trial.^[^ ^11^, ^29^ ^]^ Interestingly, combination strategies related to MEK inhibition are also being tried. Previous studies have indicated that the combination of Gαq inhibitor (YM‐254890) and MEK inhibitor (trametinib) showed prolonged suppression of MAPK signaling in preclinical UM models and enhanced therapeutic efficacy in UM.^[^ ^30^ ^]^ Thus, we propose a hypothesis that our Gαq inhibitor, GQ127, could also be combined with MEK inhibitors (Figure S3B, Supporting Information) to enhance synergistic growth inhibition, thus providing a promising therapeutic strategy in UM. Conformably, our results demonstrated that the combined treatment with selumetinib and GQ127 significantly inhibited cell proliferation (Figure 3K; Figure S3C, Supporting Information). Notably, the combined treatment significantly decreased the expression of VGF and the phosphorylation of ERK and CREB (Figure 3L; Figure S3D, Supporting Information). Next, we established a xenograft model of MP41 cells in nude mice to further determine the therapeutic benefit of GQ127 in combination with selumetinib. The results showed that the combination of GQ127 and selumetinib significantly inhibited tumor growth in the UM xenograft model compared with the vehicle and single reagent treated groups, with about 70% tumor growth inhibition (TGI) (Figure 3M–O). Changes in protein levels of VGF, p‐CREB, and p‐ERK in xenograft tumor tissues were consistent with the cell experiments in vitro (Figure 3P). Besides, no significant changes in body weights or obvious signs of toxicity were observed (Figure S3E,F, Supporting Information). Therefore, our results suggested that combined inhibition of Gαq and MEK significantly reduced tumor burden both in vitro and in vivo, thus providing an effective therapeutic strategy for advanced UM.

### 2.4 VGF Promotes Migration and Invasion of Gαq Mutant Uveal Melanoma Cells

**Figure 4:**
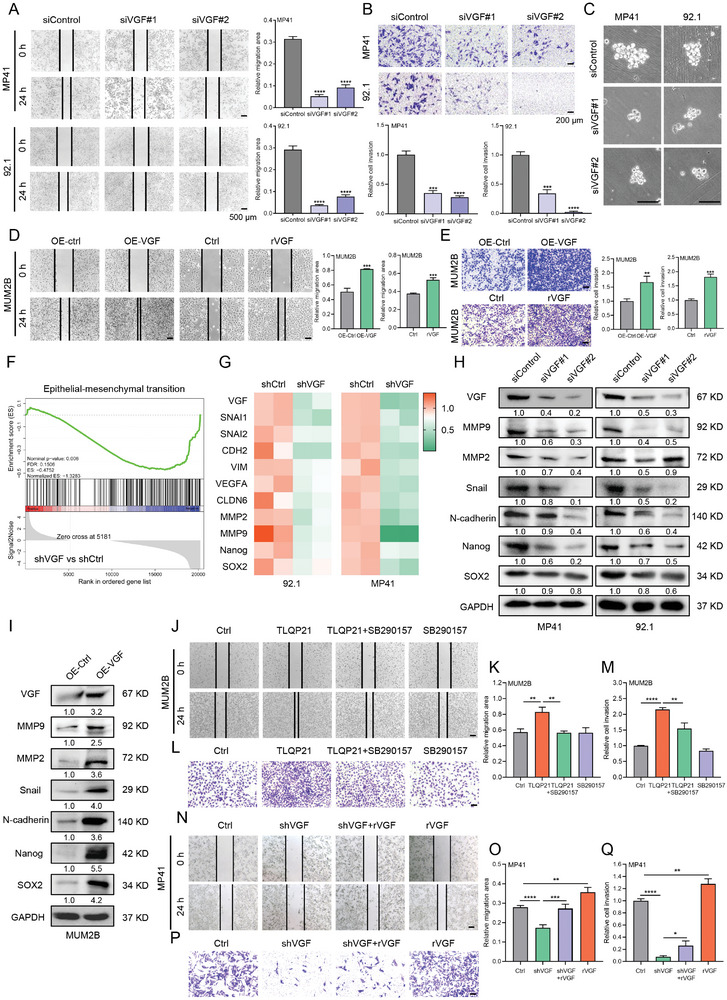
VGF promotes migration and invasion of Gαq mutant uveal melanoma cells. A) Cell migration determined by wound healing assay in UM cells treated with siRNAs against VGF or control siRNA. Scale bars = 500 µm. Right: quantification of cell migration (Fold change). Data were presented as mean ± SD, *n* = 3. ****p < 0.0001 by using two‐tailed unpaired Student *t*‐test. B) Cell invasion determined by transwell assay in UM cells treated with siRNAs against VGF or control siRNA. Scale bars = 200 µm. Bottom: quantification of cell invasion (Fold change). Data were presented as mean ± SD, *n* = 3. ***p < 0.001, ****p < 0.0001 by using two‐tailed unpaired Student *t*‐test. C) Cell stemness determined by spheroid formation assay in UM cells treated with siRNAs against VGF or control siRNA for 7 d. Scale bars = 200 µm. D) Cell migration determined by wound healing assay in MUM2B cells treated with control, VGF overexpression or rVGF (100 ng mL^−1^). Left: scale bars = 500 µm; right: scale bars = 200 µm. Right: quantification of cell migration (Fold change). Data were presented as mean ± SD, *n* = 3. ***p < 0.001 by using two‐tailed unpaired Student *t*‐test. E) Cell invasion determined by transwell assay in MUM2B cells treated with control, VGF overexpression or rVGF (100 ng mL^−1^). Scale bars = 200 µm. Right: quantification of cell invasion (Fold change). Data were presented as mean ± SD, *n* = 3. **p < 0.01, ***p < 0.001 by using two‐tailed unpaired Student *t*‐test. F) GSEA analysis indicated a significant change in EMT signaling pathway in VGF knockdown cells. G) Validation of the mRNA expression of the genes involved in the EMT pathway and stemness pathway in shCtrl and shVGF by qRT‐PCR. H) Immunoblotting of the indicated proteins in UM cells treated with siControl and siVGF (#1 and #2) for 72 h. I) Immunoblotting of the indicated proteins in MUM2B cells treated with control or VGF overexpression for 72 h. J,K) Cell migration determined by wound healing assay in MUM2B cells treated with vehicle, TLQP21 (10 × 10^−6^ m), and SB290157 (4 × 10^−6^ m) for 24 h. Scale bars = 500 µm. Right: quantification of cell migration (Fold change). Data in (K) were presented as mean ± SD, *n*  =  3. **p < 0.01 by using two‐tailed unpaired Student *t*‐test. L,M) Cell invasion determined by transwell assay in MUM2B cells treated with vehicle, TLQP21 (10 × 10^−6^ m), and SB290157 (4 × 10^−6^ m) for 24 h. Scale bars = 200 µm. Right: quantification of cell invasion (Fold change). Data in (M) were presented as mean ± SD, *n*  =  3. **p < 0.01, ****p < 0.0001 by using two‐tailed unpaired Student *t*‐test. N,O) Cell migration determined by wound healing assay in MP41 cells treated with Ctrl, shVGF, and rVGF (100 ng mL^−1^) for 24 h. Scale bars = 500 µm. Right: quantification of cell migration (Fold change). Data in (O) were presented as mean ± SD, *n*  =  3. **p < 0.01, ***p < 0.001, ****p < 0.0001 by using two‐tailed unpaired Student *t*‐test. P,Q) Cell invasion determined by transwell assay in MP41 cells treated with Ctrl, shVGF, and rVGF (100 ng mL^−1^) for 24 h. Scale bars = 200 µm. Right: quantification of cell invasion (Fold change). Data in (Q) were presented as mean ± SD, *n*  =  3. *p < 0.05, **p < 0.01, ****p < 0.0001 by using two‐tailed unpaired Student *t*‐test.

To investigate the pro‐metastasis role of VGF in UM cells, we next performed wound healing assay and transwell cell invasion assay in UM cells with VGF knockdown or overexpression. Knockdown of VGF significantly reduced the migration and invasion ability of Gαq mutant UM cells (**Figure** **4**A,B). In contrast, overexpression of VGF remarkably promoted cell migration and invasion (Figure 4D,E). Importantly, rVGF treatment also effectively increased the migration and invasion ability of UM cells. Furthermore, knockdown of VGF remarkably inhibited spheroid formation in Gαq mutant UM cells (Figure 4C). In addition, the phenomenon of diminished migration, invasion, and spheroid formation was also notably observed in the potential metastatic UM cell line OMM2.3 (Figure S4A–C, Supporting Information), which was derived from liver metastases of UM patients and harbors GNAQ mutations. Furthermore, RNA sequencing and gene set enrichment analysis (GSEA) revealed that the DEGs in VGF knockdown cells were involved in EMT signaling pathway (Figure 4F). Subsequent qRT‐PCR analysis confirmed that the genes involved in the EMT pathway and stemness pathway were downregulated in response to VGF knockdown in Gαq mutant UM cells (Figure 4G). Furthermore, we found that the protein level of the EMT marker N‐cadherin, snail, MMP2 and MMP9, and the stemness markers Nanog and SOX2 were generally decreased in VGF knockdown cells (Figure 4H; Figure S4D, Supporting Information), while overexpression of VGF promoted the expression of these markers in UM cells (Figure 4I). In addition, the VGF‐derived peptide TLQP‐21 also partly promoted cell migration and invasion, whereas C3aR inhibitor SB290157 attenuated the effect of TLQP‐21 (Figure 4J–M). Similarly, we demonstrated that external VGF could partly restore the migration and invasion ability after the knockdown of VGF in UM cells (Figure 4N–Q). Collectively, these results suggest that VGF plays a critical role in the migration, invasion and stemness of UM cells.

### 2.5 VGF Enhanced Liver Metastatic Colonization of Uveal Melanoma In Vivo

**Figure 5:**
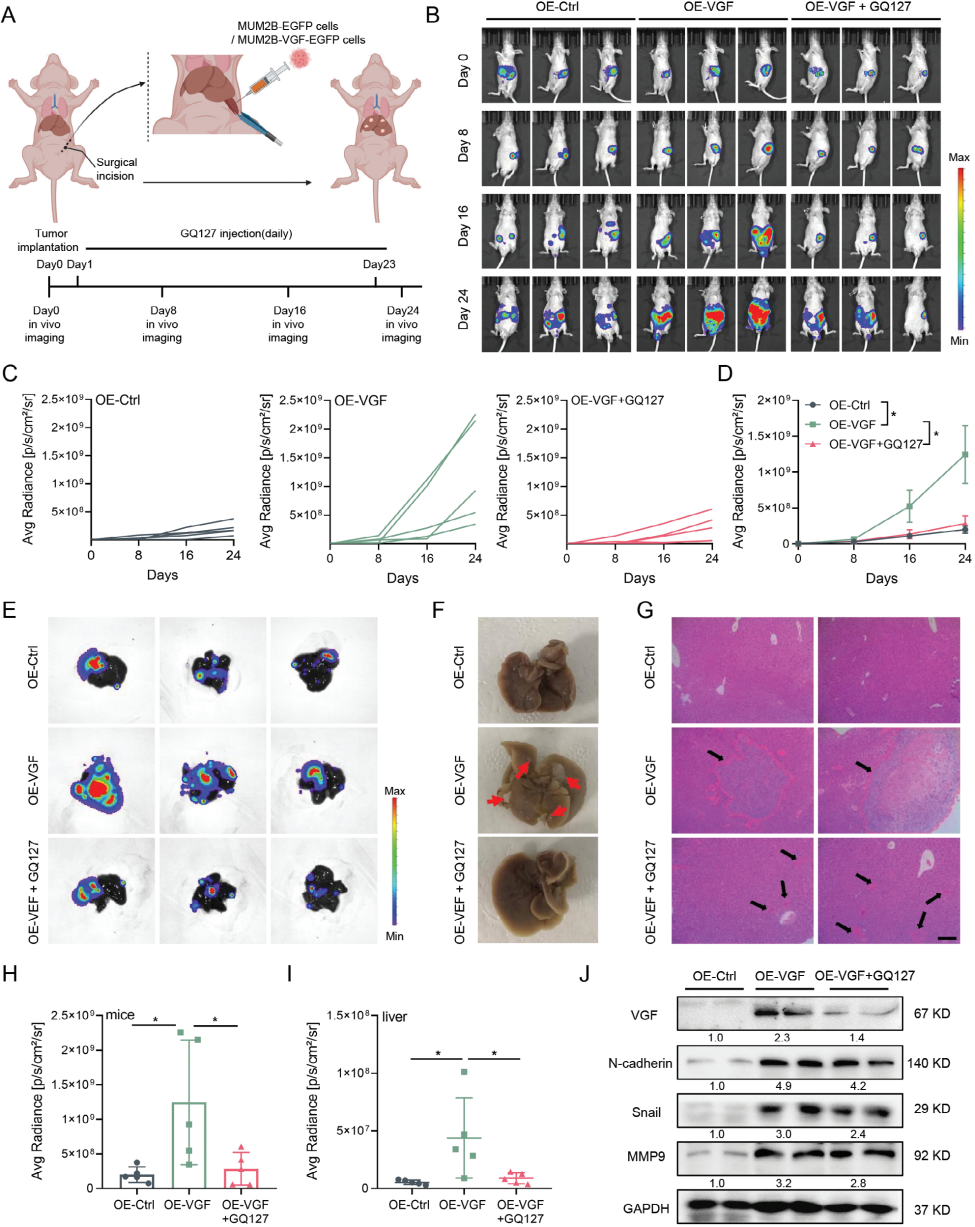
VGF enhanced liver metastatic colonization of uveal melanoma in vivo. A) Schematic illustration of the establishment and treatment of UM liver metastasis mouse model. This figure was created with BioRender.com, an online platform for data analysis, with the agreement number is HL275Q4JWE. B) MUM2B‐EGFP and MUM2B‐VGF‐EGFP cells were intrasplenically injected into BALB/c‐nu/nu mice as in (A) (*n* = 5 per group). Representative bioluminescent images of hepatic metastasis mouse model on 24 d are shown. C,D) Quantification for bioluminescence in 24 d are shown. Data in (D) were presented as mean ± SEM, *n*  =  5. *p < 0.05 by using two‐tailed unpaired Student *t*‐test. E) The representative bioluminescent images of the liver on day 24. F) Representative images of livers fixed in paraformaldehyde on day 24. Liver metastases were shown with red arrows. G) HE staining in liver paraffin section from each group. Scale bars = 100 µm. Liver metastases were shown with black arrows. H) Quantification for bioluminescence of mice in day 24 are shown. Data were presented as mean ± SEM, *n*  =  5. *p < 0.05 by using two‐tailed unpaired Student *t*‐test. I) Quantification for bioluminescence of liver in day 24 are shown. Data were presented as mean ± SEM, *n*  =  5. *p < 0.05 by using two‐tailed unpaired Student *t*‐test. J) Immunoblotting of the indicated proteins in UM liver metastasis model.

Given that liver metastasis is the leading cause of death in UM patients and there is no effective treatment against it, we next investigate that whether VGF drives the occurrence and progression of UM liver metastasis. We established a liver metastasis model of UM in nude mice (**Figure** **5**A). Overexpression of VGF significantly exacerbated liver‐metastatic burden after intrasplenic injection of the cancer cells into mice (Figure 5B–D,H), whereas our Gq inhibitor GQ127 appreciably attenuated the tumor burden promoted by VGF expression (Figure 5B–D). Noticeably, overexpression significantly increased the number of metastatic nodules on the liver surface (Figure 5F), as well as the bioluminescence signal intensity of liver tumor burdens (Figure 5E,I), which was partly reversed by GQ127 treatment.

Histologic examination indicated a remarkable increase in the number and size of the metastatic nodules in the livers of VGF overexpression mice (Figure 5G), compared to those in vehicle‐treated mice. In addition, GQ127 significantly inhibited the metastatic nodules driven by VGF (Figure 5G). Furthermore, we isolated tumor section of liver metastases tissue, then used them for protein extraction. The immunoblotting results showed that VGF overexpression increased the protein level of VGF and EMT‐related marker in vivo, while GQ127 treatment partially rescue the effects (Figure 5J). These results demonstrated a pro‐metastatic role of VGF in UM, suggesting that VGF may be a promising biomarker and target for metastatic UM patients.

### 2.6 VGF Promotes UM Growth and Metastasis by Regulating TGF‐β‐SMAD Signaling Pathway

**Figure 6:**
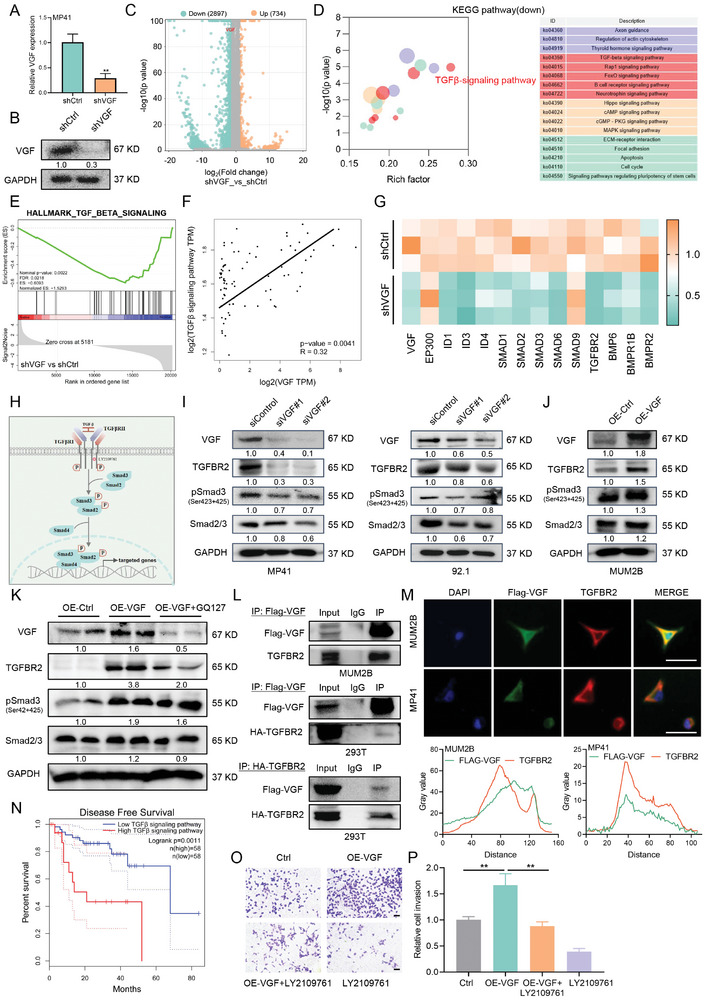
VGF promotes uveal melanoma growth and metastasis by regulating TGF‐β‐SMAD signaling pathway. A) The knockdown effect of VGF by shRNA in MP41 cells was determined by qRT‐PCR analysis. Data were presented as mean ± SD, *n* = 3. **p < 0.01 by using two‐tailed unpaired Student *t*‐test. B) The knockdown effect of VGF by shRNA in MP41 cells was determined by immunoblotting analysis. C) Volcano plot of the DEGs between shVGF and shCtrl in MP41 cells detected by RNA seq. Fold change [FC] > 2, *p* < 0.05. D) Down‐regulated pathway of VGF knockdown in MP41 cells detected by RNA seq. E) GSEA analysis of TGF‐β signaling pathway between shVGF and shCtrl in MP41 cells detected by RNA seq. F) The correlation analysis of VGF and TGF‐β signaling pathway in UM patients from UVM‐TCGA database. G) Validation of the mRNA expression of the genes involved in the TGF‐β signaling pathway in shCtrl and shVGF by qRT‐PCR. *n* = 3. H) Schematic illustration of the canonical TGFβ/SMAD signaling pathway. The red circle represents the small molecule inhibitor of TGF‐β receptors I and II, LY2109761. I) Immunoblotting of the indicated proteins in UM cells treated with siControl and siVGF (#1 and #2) for 72 h. J) Immunoblotting of the indicated proteins in MUM2B cells with control or VGF overexpression for 72 h. K) Immunoblotting of the indicated proteins in UM liver metastasis model. L) Co‐IP showing the binding of VGF and TGFBR2 in MUM2B cells (top). Co‐IP showing the binding of VGF and TGFBR2 in HEK‐293T cells (bottom). M) Representative images and quantification of immunofluorescence colocalization of Flag‐VGF (green) and TGFBR2 (red) in MUM2B (top) and MP41 (bottom) cells. Scale bars = 50 µm. *n* = 3. N) The disease‐free survival analysis of UM patients from TCGA project with TGF‐β signaling high or low expression levels (defined by RNA sequencing with group cutoff in high‐25% (*n* = 58)/low‐75% (*n* = 58)) in UVM‐TCGA database. O) Cell invasion detected by transwell assay in MUM2B cells treated with OE‐Ctrl, OE‐VGF, and LY2109761(5 × 10^−6^ m) for 24 h. Scale bars = 200 µm. P) Quantification of cell invasion (Fold change). Data were presented as mean ± SD, *n* = 3. **P < 0.01 by using two‐tailed unpaired Student *t*‐test.

To comprehensively delineate the underlying molecular mechanisms by which VGF modulates UM progression and metastasis, we performed transcriptome sequencing analysis in MP41 cells after stable knockdown of VGF (shVGF) (**Figure** **6**A,B). We identified 734 up‐regulated and 2897 down‐regulated genes in MP41 cells with VGF knockdown (Figure 6C). Kyoto Encyclopedia of Genes and Genomes (KEGG) pathway enrichment analysis of RNA‐seq identified the transforming growth factor‐β (TGF‐β) signaling pathway as one of the most significantly downregulated pathways in the VGF‐knockdown cells (Figure 6D). GSEA analysis also revealed that the TGF‐β signaling pathway was downregulated in the VGF knockdown group (Figure 6E). Furthermore, the expression of VGF is positively associated with the TGF‐β signaling pathway signature in UM tumors (Figure 6F). We performed qRT‐PCR analysis and confirmed that the genes involved in the TGF‐β signaling pathway were downregulated generally in response to VGF knockdown in MP41 cells (Figure 6G). The TGF‐β signaling is an important regulator of metastasis pathways in many types of cancer, including melanomas, gliomas, and breast cancer.^[^ ^31^ ^]^ The TGF‐β signaling pathway is initiated by the binding of TGF‐β to TGFBR2, which recruits and phosphorylates TGFBR1. In turn, activated TGFBR1 phosphorylates SMAD2/3, recruiting SMAD4 to form hetero‐oligomers with phosphorylated SMAD2/3, and then regulate the expression of target genes in the nucleus (Figure 6H).^[^ ^32^ ^]^

To verify the function of VGF in TGF‐β signaling, we next constructed VGF knockdown in both MP41 and 92.1 cells. Immunoblotting analysis revealed that VGF knockdown reduced the expression of TGFBR2 and SMAD3 phosphorylation in Gαq mutant UM cells in the presence of TGF‐β (Figure 6I). In contrast, VGF overexpression slightly increased TGFBR2 and phospho‐SMAD3 protein expression in UM cells in the presence of TGF‐β (Figure 6J). We also detect the protein expression levels of TGF‐β/SMAD signaling related genes in UM liver metastasis model, and observed that VGF overexpression increased the protein level of TGFBR2 and phospho‐SMAD3 in vivo (Figure 6K). As previous studies have suggested that interactions of TGFBR2 with other proteins that contribute significantly to the stability of the receptors and the diverse downstream signaling, we questioned whether VGF interacted with TGFBR2 in UM. We performed co‐immunoprecipitation (Co‐IP) and verified that VGF interacted with TGFBR2 in UM cells (Figure 6L) and HEK‐293T cells (Figure 6L). Immunofluorescence colocalization further confirmed that VGF binds to TGFBR2 in UM cells (Figure 6M).

We next examined the clinical prognostic impact of TGF‐β signaling in TCGA patients with UM. Higher levels of TGF‐β signaling were associated with poorer disease‐free survival in patients with UM (Figure 6N). To investigate whether TGFBR2 mediated VGF‐dependent metastasis, we used LY2109761(Figure 6H), a small molecule inhibitor of TGF‐β receptor I and II,^[^ ^33^, ^34^ ^]^ which inhibit the pathway at the level of receptor activation, in VGF stable overexpression UM cells. We found that inhibition of TGFBR2 alleviated the facilitating effects on cell invasion mediated by VGF overexpression (Figure 6O–P). In addition, we performed TGFBR2 overexpression in VGF stable knockdown UM cells (Figure S5A, Supporting Information). Our results revealed that TGFBR2 partly alleviated the cell invasion inhibition by VGF‐knockdown in MP41 cells (Figure S5B, Supporting Information). All the same, the role and interaction mechanism of TGFBR2 and VGF in driving UM metastasis are still worthy of further study. Collectively, these results superficially suggested that VGF regulates TGF‐β‐SMAD signaling by directly binding to TGFBR2 to promote the progression and metastasis of UM.

### 2.7 VGF Triggers Fibrosis and Activation of HSCs to Promote UM Liver Metastasis

Several studies have revealed that HSCs are one of the important fibrogenic cell types in liver fibrosis, and may facilitate liver metastasis by remodeling liver premetastatic niche.^[^ ^35^ ^]^ Given our previous findings that VGF regulates the TGF‐β signaling pathway, which is closely associated with fibrosis,^[^ ^36^ ^]^ we next investigate whether VGF promotes colonization in the liver of UM cells through the activation of HSCs.

**Figure 7:**
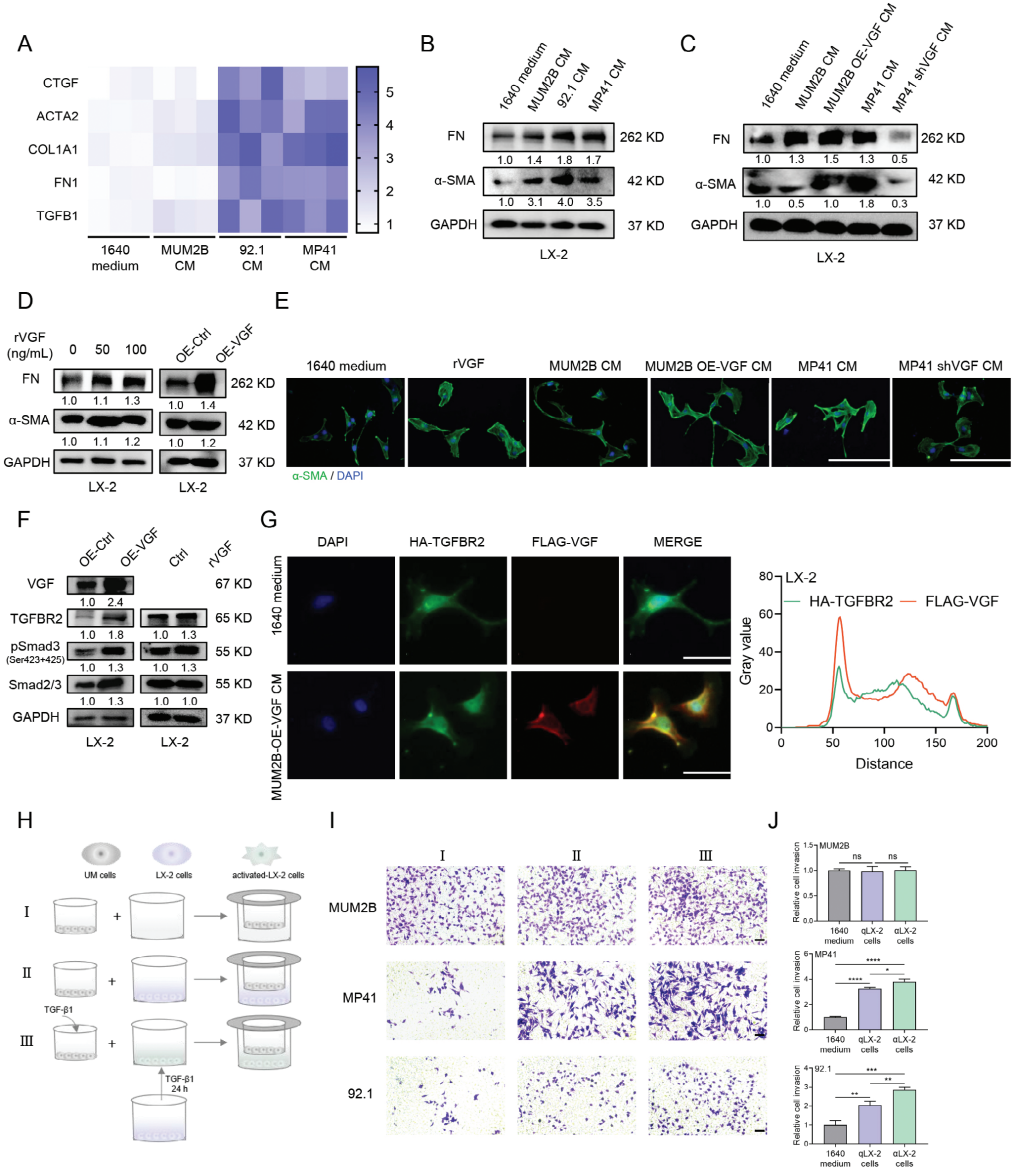
VGF triggers fibrosis and activation of HSCs to promote uveal melanoma hepatic metastasis. A) qRT‐PCR analysis of the fibrosis related genes in LX‐2 cells exposed to 1640 medium, MUM2B CM, 92.1 CM or MP41 CM. *n* = 3. B) Immunoblotting analysis of α‐SMA and FN in LX‐2 cells exposed to 1640 medium, MUM2B CM, 92.1 CM or MP41 CM for 48 h. C) Immunoblotting analysis of α‐SMA and FN in LX‐2 cells exposed to 1640 medium, MUM2B CM, MUM2B‐VGF CM, MP41 CM or MP41‐shVGF CM for 48 h. D) Immunoblotting analysis of α‐SMA and FN in LX‐2 cells treated with VGF overexpression for 72 h or rVGF(0, 50, 100 ng mL^−1^) for 24 h. E) Immunofluorescence analysis of α‐SMA of LX‐2 cells exposed to 1640 medium, rVGF, MUM2B CM, MUM2B‐VGF CM, MP41 CM, or MP41‐shVGF CM for 48 h. Representative images were shown. Scale bars = 200 µm. *n* = 3. F) Immunoblotting analysis of the indicated proteins in LX‐2 cells treated with VGF overexpression for 72 h or rVGF(0, 100 ng mL^−1^) for 24 h. G) Representative images (left) and quantification (right) of immunofluorescence colocalization of HA‐TGFBR2 (green) and Flag‐VGF (red) in LX‐2 cells exposed to 1640 medium or MUM2B‐Flag‐VGF CM. Scale bars = 50 µm. *n* = 3. H) Schema of co‐cultured UM cells with LX‐2 cells. I, monocultured UM cells with RPMI‐1640 medium in Transwell systems; II, coculture UM cells with qLX‐2 cells in Transwell systems. Upper: UM cells; lower: unactivated LX‐2 cells. III, coculture UM cells with αLX‐2 cells in Transwell systems. Upper: UM cells; lower: activated LX‐2 cells. For LX‐2 activation, LX‐2 cells were treated with TGF‐β (10 ng mL^−1^) for 24 h. I) Transwell cell invasion for UM cells as indicated approaches (H). Scale bars = 200 µm. J) Quantification of cell invasion (Fold change). Data were presented as mean ± SD, *n* = 3. ns, not significant. Ns, *p < 0.05, **p < 0.01, ***p < 0.001, ****p < 0.0001 by using two‐tailed unpaired Student *t*‐test.

Interestingly, qRT‐PCR and immunoblotting analysis revealed that the CM obtained from Gαq mutant UM cells (MP41 and 92.1), which also are VGF‐riched, rather than that from MUM2B cells, significantly increased the expression of the fibrosis related marker alpha‐smooth muscle action (α‐SMA) and FN of LX‐2 cells (**Figure** **7**A,B). We observed that LX‐2 cells were activated by the stimulation of CM obtained from MP41 cells and VGF‐overexpressed MUM2B cells (Figure 7C). Moreover, overexpression of VGF or rVGF treatment also increased the protein level of α‐SMA and FN in LX‐2 cells (Figure 7D). The immunofluorescent staining of α‐SMA further confirmed this activation of LX‐2 cells in VGF‐riched CM or rVGF treatment (Figure 7E). These results suggested that VGF activates HSCs and might provide a favorable microenvironment for liver metastasis in UM.

Immunoblotting analysis of whole‐cell lysates of LX‐2 cells showed that overexpression of VGF or rVGF treatment also slightly increased the expression of TGFBR2 and the phosphorylation of SMAD3 (Figure 7F). Considering that VGF is a secreted protein and could stimulate HSCs activation, we wondered whether VGF secreted from UM cells binds to TGFBR2 in HSCs. Furthermore, we generated the forced expression of TGFBR2 in LX‐2 cells (Figure S5C, Supporting Information), and then exposure of LX‐2 cells (OE‐HA‐TGFBR2) to the CM prepared from MUM2B cells with Flag‐VGF overexpression. Immunofluorescence staining showed that the VGF secreted by UM cells also interacted with TGFBR2 expressed in LX‐2 cells (Figure 7G), suggesting that VGF may also activate HSCs via the TGF‐β‐SMAD signaling pathway through paracrine loops.

Previous studies revealed that cancer cells induce the activation of HSCs,^[^ ^37^ ^]^ and the activated HSCs further make feedback to enhance tumor growth and metastasis in the process of metastasis.^[^ ^38^ ^]^ We then explore whether activated HSCs trigger the growth of UM cells. Exposure of UM cells to the culture environment of LX‐2 cells (inactivated, named qLX‐2 or activated, named αLX‐2, which was pretreated with TGF‐β for 24 h) in transwell‐coculture system, as illustrated in Figure 7H. The invasion effect of MP41 and 92.1 cells cocultured with qLX‐2 and αLX‐2 cells was enhanced, while the effect of cocultured with αLX‐2 cells was stronger (Figure 7I–J). By contrast, this treatment did not induce such a facilitated effect on MUM2B, a Gq wide type UM cell line (Figure 7I–J).

Overall, our results suggest that VGF triggers the activation of HSCs through an autocrine and paracrine loop, possibly via the TGF‐β‐SMAD signaling pathway, and the activated HSCs further make feedback to enhance the ability of Gαq mutant UM cell invasion, thus facilitating liver metastatic colonization of UM.

## 3 Discussion

UM is a malignant tumor deriving from extracutaneous melanocytes residing within the uveal tract of the eye.^[^ ^39^ ^]^ Treatment of primary UM with radiotherapy, enucleation or other modalities achieves local control in more than 90% of patients, whereas 40% or more ultimately develop distant metastases, most commonly in the liver (93%).^[^ ^40^, ^41^ ^]^ These patients with metastatic UM have historically had a dismal prognosis, with a median overall survival duration of about 1 year,^[^ ^42^ ^]^ owing to the limited efficacy of available local and systemic treatment. Until January 2022, tebentafusp, a bispecific T cell‐engager targeting CD3 and glycoprotein 100 (gp100) in an HLA‐A*02:01‐restricted fashion,^[^ ^43^ ^]^ became the first systemic therapy to achieve regulatory approval for metastatic UM. This advance has offered a new and promising prospect for this disease. Nevertheless, the effective approach for UM is very limited and it is urgent to explore the mechanism of advanced UM and discover a new therapeutic strategy.

The neurosecretory protein VGF plays an important role in neurodegenerative and psychiatric diseases, but its role in cancer remains unclear. In the present study, we report the role of tumor secreted VGF in promoting the growth and liver metastatic colonization of UM, through an autocrine and paracrine loop. We are the first to demonstrate that VGF secretion and expression are upregulated in Gαq mutant UM based on bioassay, secretomics, and transcriptome sequencing. Higher VGF levels were associated with poorer clinical prognosis, making it a promising predictor of survival in UM patients. Sincerely, we tried to consider Gq mutation status in survival analysis, which inflate the importance of VGF expression more scientifically. Unfortunately, these datasets, which were used for survival analysis in our study, mainly included patient clinical data or patient characteristics, but did not include Gq mutation status. As a result, we are unable to perform further analysis of survival data based on Gq mutation status. We will continue to consider this suggestion in future studies. Moreover, the knockdown of VGF inhibited cell growth, proliferation, survival and promoted cell apoptosis in Gαq mutant UM cells. Moreover, intratumoral injection of siRNA targeting VGF significantly inhibited the growth of UM xenografts. In our previous work, we developed a novel selective Gq inhibitor, GQ127, and investigated its antitumor efficacy and mechanisms in UM.^[^ ^23^ ^]^ Importantly, a combination of siVGF and Gq inhibitor GQ127 almost totally blocked the growth of UM xenografts, and the tumor shrunk to a size less than the initial volume. Also of note, as a secretory factor, VGF acts on tumor as well as nontumor cells. Despite our initial findings that knockdown of VGF significantly inhibited the growth of UM cells, whereas its impact on nontumor cells was comparatively limited, more evidence is needed to comprehensively evaluate the safety and efficacy of VGF inhibition in the treatment of malignant tumors in the future.

To elucidate the molecular mechanism by which Gαq regulates VGF in UM, we used RNA‐seq analyses to identify the potential signaling pathway. Based on the enrichment analysis, we focused on the MAPK signaling pathway, which is one of the downstream signaling cascades activated by GNAQ and GNA11 mutations in UM.^[^ ^44^ ^]^ Our results demonstrated that knockdown of GNAQ inhibited the phosphorylation of ERK and CREB in UM cells and xenografts. Recent studies have reported that CREB, a transcription factor dependent on the MAPK signaling pathway, is involved in the regulation of VGF expression.^[^ ^25^ ^]^ Consistent with the reports, our results also demonstrate that CREB transcriptionally regulates VGF expression by directly binding to the DNA response element of the VGF gene, indicating that GNAQ regulates VGF expression through MAPK/CREB signaling in UM. Recent studies have shown that inhibition of MEK has preclinical efficacy consistent with the constitutive MAPK pathway signaling resulting from upstream activating GNAQ, GNA11 alterations in UM.^[^ ^45^ ^]^ However, there was no improvement in overall survival in several clinical trials evaluating selumetinib, an oral and potent inhibitor of MEK1/2, in patients with UM.^[^ ^11^, ^29^, ^46^ ^]^ Meanwhile, combination drug strategies related to MEK inhibition are also being tried and implemented. Notably, a combination with Gαq inhibitor YM‐254890 and MEK inhibitor showed synergistic growth inhibition and tumor shrinkage.^[^ ^30^ ^]^ Inspired by this, we combined our Gq inhibitor GQ127 with MEK inhibitor selumetinib, and demonstrated that the combination effectively improves therapeutic efficacy in UM, providing a promising and effective treatment approach in combination regimens.

Clinically, liver metastasis is the main challenge for UM patients, due to limited treatment. Decisions on the treatment need to precede the occurrence of fatal metastasis. In the study, we found that VGF is essential for the metastasis of UM in vitro and in vivo. Knockdown of VGF significantly suppressed the migration, invasion, spheroid formation and the expression of EMT‐related genes in Gαq mutant UM cells. In contrast, overexpression of VGF increased the ability of metastasis in UM cells. Furthermore, we established a liver metastasis model of UM in nude mice, and demonstrated that VGF overexpression conspicuously promoted metastatic tumor burdens in the liver, suggesting that VGF potently drives liver metastatic colonization of UM. Moreover, the Gq protein inhibitor GQ127 treatment appreciably attenuated the tumor burdens. Complementally, we found that there were no other metastatic sites besides the liver and spleen. Previous studies have revealed that HSCs are considered liver‐specific pericytes and play a crucial role in the pathological process of liver metastasis in several cancers, including colorectal cancer,^[^ ^47^ ^]^ pancreatic cancer^[^ ^48^ ^]^ and UM.^[^ ^49^ ^]^ Cancer cells may secrete multiple growth factors to activate HSCs,^[^ ^37^ ^]^ which in turn make a positive feedback to enhance tumor growth, metastasis, angiogenesis and immune escape.^[^ ^38^ ^]^ Interestingly, our results revealed that VGF, either overexpression, given directly or derived from CM of UM cells, could activate HSCs, thus creating a favorable microenvironment for UM liver metastasis. Meanwhile, the activated HSCs enhanced the ability of Gαq mutant UM cell invasion, thus facilitating liver metastatic colonization of UM.

Using transcriptome sequencing analysis, we identified the TGF‐β signaling pathway as a key downstream pathway in driving UM progression and metastasis. It was reported that TGF‐β signaling plays a critical role in medically relevant processes of immunity, inflammation, cancer, and fibrosis.^[^ ^50^ ^]^ And TGFβ receptors control the expression of hundreds of genes involved in a pathway related to apoptosis, cell cycle, angiogenesis, and EMT through the canonical SMAD pathway. Here, our results showed that the knockdown of VGF remarkably downregulated the phosphorylation of SMAD3 and the expression of TGFBR2, while overexpression of VGF promoted the activation of SMAD3 and the protein level of TGFBR2, indicating that VGF may regulate the target genes of the canonical TGF‐β/SMAD pathway. Previous studies reported that monosomy for chromosome 3 is common in uveal melanoma.^[^ ^51^ ^]^ Interestingly, TGFBR2 is located on chromosome 3p22. TGFBR2 is an important receptor mediating the activation of the canonical TGFβ/SMAD signaling pathway, acting as a gatekeeper for downstream signal activation. We used Flag‐VGF and HA‐TGFBR2 to perform a Co‐IP study, and found that VGF interacted with TGFBR2 both in UM cells and 293T cells. Consistently, the subsequent immunofluorescence results confirmed that VGF could bind to TGFBR2 in UM cells. This suggested that VGF may be a partner of TGFBR2. Considering that VGF is a secreted protein that can stimulate HSCs activation, we wondered whether VGF secreted from UM cells binds to TGFBR2 in HSCs. Furthermore, overexpression of Flag‐VGF in UM cells and overexpression of HA‐TGFBR2 in HSCs was performed, and then exposure of the HSCs to the CM prepared from UM cells. The subsequent immunofluorescence staining showed that the VGF secreted by UM cells also interacted with TGFBR2 expressed in LX‐2 cells. In addition, whether HSCs exposed to rVGF or direct overexpression of VGF in HSCs both promote SMAD3 phosphorylation. This indicated that tumor‐secreted VGF may interact with TGFBR2 of HSCs and then activate the canonical TGF‐β‐SMAD signaling pathway to trigger liver fibrosis.

The analysis of the tumor tissue and biopsy from UM patients may provide more evidence for the role of VGF in UM progression, but we did not get enough tumor tissue at the current stage. Furthermore, it is undeniable that healthy melanocytes could be the most suitable nontumor control for UM compared to all the other eye cell types. Notwithstanding these limitations, this study does suggest the potential of VGF as a promising biomarker and a driver gene in the progression and metastasis of UM. A better understanding of the underlying mechanisms of metastatic UM, the search for biomarkers for early diagnosis and novel therapeutic strategies to alleviate or overcome UM liver metastases, will provide hope for continued progress in the future. This study advances our understanding of the complicated metastatic process of UM and may provide new targets and treatment strategies for metastatic UM.

## 4 Experimental Section

### Cell Line and Cell Culture

MP41 (GNA11^Q209L^) cell line was offered by Professor Jingxuan Pan in the State Key Laboratory of Ophthalmology, Zhongshan Ophthalmic Center, Sun Yat‐sen University. HEK293T, LX‐2, ARPE‐19, and HLE‐B3 cell lines were purchased from the American Type Culture Collection (ATCC, USA). MUM2B (GNAQ and GNA11^WT^), and 92‐1 (GNAQ^Q209L^) cell lines were purchased from Shanghai FuHeng Biology Co., Ltd. The OMM2.3 cell line was purchased from Biobw. All the cell lines were tested and authenticated by short tandem repeat (STR) matching analysis of cells. MP41, OMM2.3, 92.1, and MUM2B cells were cultured in RPMI 1640 medium (Gibco, USA) supplemented with 10% fetal bovine serum (FBS, Gibco, USA) and 1% penicillin–streptomycin (P.S, Gibco, USA). HEK293T, LX‐2, ARPE‐19, and HLE‐B3 cells were cultured in DMEM (Gibco, USA) containing 10% FBS and 1% Penicillin–Streptomycin. All cells were cultured at 37 °C under 5% CO_2_. Cell culture plates and dishes were purchased from NEST Biotechnology, China.

### Reagents, Peptides, Recombinant Proteins, and Antibodies

Selumetinib (AZD6244) was purchased from Selleck (S1008, USA). SB290157 was from Selleck (S8931, USA). LY2109761 was from TargetMol (T2123, USA). TLQP‐21, peptide sequence TLQPPSALRRRHYHHALPPSR were synthesized by Sangon Biotech (China). rVGF was from Cloud‐clone (APB166Hu61, USA). Anti‐VGF (WB 1:100, ab69989), Anti‐phospho S423+S425‐SMAD3 (WB 1:1000, ab52903), and Anti‐FN (WB: 1:1000, ab2413) were from Abcam (UK). Anti‐ERK1/2 (WB 1:1000, #4348), Anti‐GAPDH (WB 1:1000, #5174), Anti‐phopho202/204‐ERK1/2 (WB 1:1000, #5174), Anti‐CREB (WB 1:1000, #9197), Anti‐phopho133‐CREB (WB 1:1000, #9198), and Anti‐SMAD2/3 (WB 1:1000, #8685) were purchased from Cell Signaling Technology (USA). Anti‐α‐SMA (WB 1:1000; IF 1:500; GB11136) and Anti‐Ki67 (IHC 1:500, GB111141) were from Servicebio (China). Anti‐Flag‐tag (IP 2ug; WB 1:1000; IF: 1:200; 80010‐1‐RR), Anti‐HA (IP 2ug; WB 1:1000; IF: 1:50; 51064‐2‐AP) and Anti‐TGFBR2(WB 1:1000; IF 1:100; 66636‐1‐Ig) were from Proteintech (USA). Anti‐Gq (WB 1:500, sc‐365906) was from Santa cruz (USA).

### Plasmid Constructions, Cell Transfection, and Infection

For overexpression constructs, human VGF cDNA (NM_003378.4) was cloned into pEnCMV‐3×Flag vector; human CREB1 cDNA (NM_134442.5) was cloned into a pCDH‐CMV‐FLAG‐EF1a‐Puro vector; human TGFBR2 cDNA (NM_001024847.3) was cloned into pCMV‐Myc‐HA‐Neo vector.

For a stable expression system, the corresponding vector together with packing and helper plasmids PAX2 and MD2G were co‐transfected into HEK‐293T cells by Lipofectamine 2000 Transfection Kit (11668019, Invitrogen, USA) by the manufacturer's instructions. Viruses were produced, filtered, and titrated according to the manufacturer's instructions, and cells were infected with 8 mg mL^−1^ polybrene (TR‐1611003, Sigma, USA). After screening with puromycin (HY‐B1743A, MCE, USA) or G418 (ST081, Beyotime, China) in corresponding concentration for 5 d, stable cells were harvested for subsequent experiments. shRNA sequences of VGF are listed in Table S1 in the Supporting Information.

### Western Blot Analysis

Cells in a 6‐well plate or tumor tissue were lysed in RIPA buffer (P0013B, Beyotime, China) containing protease inhibitors (Beyotime, China) and phosphatase inhibitors (Bimake, USA). Protein concentrations were determined by the BCA protein assay kit (23252, ThermoFisher, USA). Proteins were separated by 8–12% SDS‐PAGE and transferred to polyvinylidene fluoride (PVDF) membranes (Millipore, USA). The membranes were blocked and blotted with related antibodies at 4 °C overnight. Secondary HPR‐conjugated antibodies were labeled at room temperature for 1 h. Lastly, the membranes were detected by chemiluminescence (1708280, Bio‐Rad, USA). The loading controls used in the WB analyses were derived from the same experiment (i.e., cell lysates) and blots were processed in parallel when it was not possible to run on the same gel. GAPDH was used as an internal control. The gray scale analysis of protein bands was performed by ImageJ software. The relative protein expression values of each group (the gray scale of target protein /GAPDH, p‐CREB/CREB or p‐ERK/ERK; fold change) were displayed below the corresponding protein bands.

### RNA Interference

Following the manufacturer's protocol, siRNA transfected UM cells in 6‐well plates using DharmaFECT (T‐2001‐03, Dharmacon, USA) to efficiently knock down GNAQ or VGF. Negative control was not complementary to any human gene. All siRNA sequences used in this study are listed in Table S1 in the Supporting Information. All the above siRNAs were synthesized by Sangon Biotech (China).

### RNA Isolation and Real‐Time Quantitative PCR (RT‐qPCR)

Total RNAs were extracted by Trizol (ThermoFisher, USA) following the manufacturer's protocol. Using the Hifair II 1st Strand cDNA Synthesis Kit (gDNA digester plus) (YEASEN, China) synthesized cDNA. Reverse Transcription‐Polymerase Chain Reaction (qPCR) was performed with Hieff UNICON Power qPCR SYBR Green Master Mix (11197ES03, YEASEN, China). GAPDH is an internal control for the normalization according to the manufacturer's protocol. All primers used in this study are listed in Table S2 in the Supporting Information. All the above primers were synthesized by Sangon Biotech (China).

### Preparation of UM‐Conditioned Medium for Biological Experiment

UM cells were grown to ≈80% confluence in 10 cm culture dishes. After washing with PBS twice, the cells were incubated in serum‐free 1640 medium at 37 °C for 24 h. The supernatant was collected, centrifuged at 1000 rpm for 10 min, filtrated by 0.22 µm filters and stored at −80 °C. For biological experiment (such as CCK8 assay and transwell invasion assay et al.), the CM was diluted 2:1 with 1640 medium (10% FBS and 1% P.S), and then cultured with another cell.

### Cell Viability and Proliferation Assay

For the CCK8 assay, cells were cultured in 96‐well plates with fresh medium. After incubation for 24 h, several concentrations of the drugs or the indicated CM were administered to cells, and incubation was continued for another 72 h. Then, Cell Counting Kit‐8 (CCK‐8, B34304, Bimake, USA) was used to measure cell proliferation at 450 nm using a microplate reader (FLUOstar, Omega, USA) after incubation at 37 °C for an additional 1–4 h until the color of untreated controls turned to orange.

For the EDU (5‐ethynyl‐2′‐deoxyuridine) assay, UM cells in 6‐well plates were cultured with corresponding EDU reagent concentration for 2 h. The culture medium was removed, and 4% paraformaldehyde was added to fix the cells for 15 min. The samples were permeated with 0.3% TritonX‐100 in PBS after washing with PBS and dyed with click additive solution (C0075S, Beyotime, China). Incubate with Hoechst 33342 at room temperature and away from light for 10 min. The images were collected with 10 × visions in EVOS FL Auto.

### Colony Formation Assay

UM cells (MUM2B: 1×10^3^ cells; MP41 or 92.1 cells: 2×10^3^ cells) were seeded into 6‐well plates cultured for 10–14 d, with the medium or the indicated CM changed as well as the compounds added every 3 d. The cells were fixed using 4% paraformaldehyde and stained with crystal violet staining solution (Beyotime, China), and the colony pictures were captured separately.

### Mammosphere Formation Assay

For the mammosphere formation assay, cells were seeded into ultralow attachment plates at a density of 1–3 × 10^4^ viable cells mL^−1^ in DMEM‐F12 (Gbico, USA) supplemented with 100× insulin, 20 ng mL^−1^ epidermal growth factor (EGF), 20 ng mL^−1^ basic fibroblast growth factor (bFGF; Sigma) and 0.4% BSA (Sigma, USA) for 5–10 d until the mammosphere became visible.

### Cell Apoptosis Assays

Cell apoptosis induced by RNA interference was detected with an Annexin V‐FITC Apoptosis Detection Kit I (BestBio, China) per the manufacturer's protocols. After trypsin activation and centrifugation, the UM cells were resuspended by 400 µL 1 × binding buffer and transferred to a new 1.5 mL EP tube. The resuspended solution was incubated with Annexin V‐FITC 2.5 µL and propidium iodide 5 µL at 4 °C for 15 and 5 min, respectively. The analysis was done on Guava easyCyte and FlowJo 7.6 software.

### Wound Healing Assay

Cells were seeded into 6‐well plates and cultured to 90% confluent after 24 h. Wounds were made in each well of 6‐well plates using pipette tips. The cells were then allowed to migrate to the stripped area for 24/48 h. Cell migration was observed under the microscope (4×, Nikon, Japan) and photographed at different time points (0 h, 24/48 h).

### Transwell Cell Invasion Assay

Cells were inoculated in Transwell Chamber (Corning, USA) with RPIM‐1640 medium of 10% FBS, and 500 µL of the same medium was added to the bottom of the Transwell chamber. After 24 h, the upper compartment medium was changed to RPIM‐1640 without FBS. The lower compartment medium was changed to 20% FBS RPIM‐1640. After incubation for another 24 h, the samples were fixed with crystal violet solution and stained. Cells were photographed under the microscope (10×, Nikon, Japan), and selected the field of view randomly for counting. The data from three experiments were collected and plotted.

### Chip‐qPCR Analysis

As previously described,^[^ ^52^ ^]^ MP41 cells were grown to 70–90% confluence in RPMI‐1640 medium in a 15 cm culture dish and were treated with GQ127 (30 × 10^−6^ m) or vehicle (DMSO) for 48 h. Cells were then fixed with 1% formaldehyde at room temperature for 10 min and washed with ice‐cold PBS. After cells were scraped off in buffer I (0.25% Triton X‐100/10 × 10^−3^ m EDTA/0.5 × 10^−3^ m EGTA/10 × 10^−3^ m Hepes, pH 6.5). Cell pellets were collected by centrifugation and washed in buffer II (200 × 10^−3^ m NaCl/1 × 10^−3^ m EDTA/0.5 × 10^−3^ m EGTA/10 × 10^−3^ m Hepes, pH 6.5). Two hundred‐microliter cell pellets were resuspended in 1 mL of lysis buffer [0.5% SDS/10 × 10^−3^ m EDTA/50 × 10^−3^ m Tris, pH 8.1/1× protease inhibitor cocktail (Roche Molecular Biochemicals)/1mg mL^−1^ 4‐(2‐aminoethyl) benzenesulfonyl fluoride] and sonicated four times for a 30 s interval of 0.5 s pulses (Fisher, model 550 Sonic Dismembrator). Cell debris was removed by centrifugation, and the chromatin solutions were diluted 5× with dilution buffer (1% Triton X‐100/2 × 10^−3^ m EDTA/150 × 10^−3^ m NaCl/20 × 10^−3^ m Tris, pH 8.1/1× protease inhibitor cocktail). Chromatin fragments were immunoprecipitated with specific antibodies overnight at 4 °C. For a 5 mL diluted chromatin solution, the following amounts of antibodies were used: 1 µL of IgG (Millipore, Massachusetts, USA) and 2 µg of CREB (#9197, Cell Signaling Technology, Boston, USA). Dynabeads TM protein G (1004D, Invitrogen, California, USA) beads were preincubated with Chromatin solution overnight in dilution buffer and washed three times in dilution buffer before use. Immunocomplexes were recovered and eluted. After reverse cross‐linking at 65 °C overnight, the DNA fragments were purified with a GeneJET Gel Extraction Kit (K0692, ThermoFisher, USA). The immunoprecipitated DNA was analyzed by real‐time PCR with SYBR Green on an iCycler instrument. Enrichment of genomic DNA was presented as the percent recovery relative to the input. All primers used in this study are listed in Table S3 in the Supporting Information. All the above primers were synthesized by Sangon Biotech (China).

### Reporter Constructs and Reporter‐Gene Assays

VGF reporter‐gene assays were performed by transfecting 293T cells with PGL3‐VGF, pCDH‐CMV‐CREB1(human)‐FLAG‐EF1a‐Puro, and pCMV‐β‐gal for normalization. The VGF mutant form (VGF Mut) contains sequences mutated from GCTGGTGTCACG to GCTGGTGGTCCG. Briefly, 293T cells seeded in 96‐well plates in phenol red‐free DMEM medium were transfected with Lipofectamine 2000 (1168019, Invitrogen, California, USA) and the indicated plasmid DNA. 24 h after transfection, transfected cells were treated with GQ127 (30 × 10^−6^ m) for another 24 h before being harvested for β‐gal and luciferase assays. The luciferase and β‐galactosidase were then analyzed with a Luciferase Assay Substrate (Promega, Madison, Wisconsin, USA) and Luminescent β‐galactosidase Detection Kit II (Clontech, San Francisco, USA). All primers used in this study are listed in Table S4 in the Supporting Information. All the above primers were synthesized by Sangon Biotech (China).

### RNA‐Seq Analysis

Total RNA was extracted by TRizol (ThermoFisher, USA) according to the manufacturer's instructions. RNase‐free DNase I to remove genomic DNA contamination. Sequencing libraries were generated using VAHTSTM mRNA‐seq V2 Library Prep Kit for Illumina following the manufacturer's recommendations and index codes were added to attribute sequences to each sample. Paired‐end sequencing of the library was performed on the HiSeq XTen sequencers (Illumina, CA). TopHat2 and HTSeq were used for RNA sequencing (RNA‐seq) data analysis with GRCh38/hg19 as the reference genome. Differential analysis of the RNA‐seq data was performed using DESeq2 analysis. Library preparation and high‐throughput sequencing were performed by Sangon Biotech (China).

### CM Collection and Secretome Analysis

Cells were grown to ≈80% confluence in 10 cm culture dishes. After washing with serum‐free medium at 37 °C for 15 min twice, the cells were incubated in serum‐free medium at 37 °C for 24 h. The CM was collected, centrifuged at 1000 rpm for 10 min, filtrated by 0.22 µm filters, and then added with protease inhibitors. The CM was concentrated with the Ultra‐15 centrifugal filter devices with the 3‐kD cutoff (Millipore, USA), and the protein concentration was determined by BCA assay.

For the denaturation of proteins, urea with a final concentration of 8M was added to the concentrated CM sample (*n* = 2). Subsequently, the sample was incubated in 2 × 10^−3^ m dithiothreitol (DTT) at 37 °C for 2 h. For alkylation of proteins, the sample was incubated in 10 × 10^−3^ m iodoacetamide (IAA) at room temperature for 40 min, followed by diluting the sample system until the final urea concentration was less than 1 m. After overnight trypsin (90057, Thermo, USA) digest at 37 °C, the reaction was quenched by the addition of trifluoroacetic acid (TFA). Each fraction was further separated on an analytical C18 column. The peptides were identified by LC‐MS/MS analysis.

### ELISA Assay

ELISA kits were used to assay the levels of human VGF (EH0769, FineTest, USA) in cell culture supernatants according to the manufacturer's instructions. Briefly, the capture antibody was pre‐coated onto 96‐well plates. Then, add 100 µL standard or sample to each well and incubate for 90 min at 37 °C, and the plates were washed two times with washing buffer. Next, a Biotin‐labeled antibody working solution was added into the wells and incubated for 60 min at 37 °C. After three washes with washing buffer, HRP‐streptavidin conjugate was incubated for 30 min at 37 °C. After five washes with washing buffer, 90 µL TMB substrate solution was added into the wells and incubate 10–20 min at 37 °C. Then, add 50 µl stop solution and the signals were read at 450 nm.

### Co‐Immunoprecipitation

Cells were lysed using a lysis buffer supplemented with protease inhibitors and phosphatase inhibitors. After centrifugation at 15 000 rpm for 15 min, the protein concentrations were measured. Immunoprecipitation was then performed with anti‐Flag or other primary antibodies by following the manufacturer's instructions. Next, the samples were incubated with protein A/G beads for 4 h at 4 °C. Then the proteins were resolved on SDS–PAGE, transferred to PVDF membranes, and analyzed with immunoblotting.

### Immunofluorescence

Cells were fixed with 4% paraformaldehyde for 15 min at room temperature. Plates were washed three times by PBS. Then cells were permeabilized with 0.3% Triton X‐100 for 15 min at room temperature, and plates were washed thrice by PBS. The samples were blocked with goat serum (Boster, China) for 1 h at room temperature, and then, incubated with Flag‐tag, HA‐tag, TGFBR2 or α‐SMA primary antibody overnight at 4 °C. Plates were washed thrice in PBS. Next, cells were incubated with Alexa‐Fluor‐conjugated secondary antibodies for 1 h in the dark. Plates were washed thrice in PBS. Then, the cells were subsequently stained with DAPI (Yeasen, China) for 10 min. Images were captured by the imaging system. Five representative fields were captured for each condition under the same exposure time.

### Immunohistochemistry

Tumor tissue slides were deparaffinized, and rehydrated through an alcohol series followed by antigen retrieval with sodium citrate buffer. Tumor sections were blocked with 5% normal goat serum with 0.1% Triton X‐100 and 3% H_2_O_2_ in PBS for 60 min at room temperature and then incubated with appropriate primary antibodies at 4 °C overnight. IHC staining was performed with horseradish peroxidase conjugates using DAB detection. Nuclei were counterstained with DAPI. Images were taken with Nikon microscopy.

### Animal Experiments

The animal experiments were approved by the Research Ethics Committee of Sun Yat‐sen University (SYSU‐IACUC‐2021‐000230, SYSU‐IACUC‐2021‐000233, SYSU‐IACUC‐2021‐000593).

For the siVGF/GQ127 subcutaneous tumor model, 4‐week‐old BALB/c‐nu/nu mice (male, weighing 16–18 g, SPF grade, certification No. SYXK(Guangdong)2016‐0112) achieved from the Gempharmatech Co, Ltd. (Nanjing, China). MP41 cells (3×10^7^ cells/mouse) were injected subcutaneously into the flank of nude mice to a form primary tumor. When the tumor reached ≈1000 mm^3^, the tumor was excised and sliced into ≈30 mm^3^ slices, which were successfully implanted into nude mice as secondary receptors. When tumor volumes reached ≈100 mm^3^, mice were randomly divided into 10 mice in each group (*n* = 10), namely vehicle control, siControl, siVGF, GQ127, and GQ127 + siVGF. For vehicle control and the GQ127 treatment group, solvent and GQ127 were intraperitoneally injected every day with a dose of 10 mg kg^−1^. For siVGF, siContorl, and GQ127+siVGF treatment group, siVGF or siContorl was blended with the RNA transfection reagent (18668‐11‐1, Engreen, New Zealand) with the ratio of 2:1 to form the transfection complexes. The transfection complexes were intratumorally injected every 3 d for a total of six times. Tumor size was measured every 3 d by vernier caliper. The laboratory animals were maintained under standard conditions and raised following the National Research Council's guide. The average tumor volume in each group was calculated based on the equation for a prolate spheroid (tumor volume = (short‐diameter)^2^ × large‐diameter × π/6) and expressed in mm^3^.

For the combination experiment subcutaneous tumor model, 4‐week‐old BALB/c‐nu/nu mice (male, weighing 16–18 g, SPF grade, certification No. SYXK(Guangdong)2021‐0029) achieved from the Experimental Animal Center of Sun Yat‐sen University (Guangdong, China). MP41 cells (3 × 10^7^ cells/mouse) were injected subcutaneously into the flank of nude mice to a form primary tumor. When the tumor reached ≈1000 mm^3^, the tumor was excised and sliced into ≈30 mm^3^ slices, which were successfully implanted into nude mice as secondary receptors. When tumor volumes reached approximately 100 mm^3^, mice were randomly divided into four groups (*n* = 6), namely vehicle, GQ127, Selumetinib, and GQ127 + Selumetinib. GQ127 was intraperitoneally injected every day with a dose of 10 mg kg^−1^, and the vehicle was replaced with the same amount of solvent. Selumetinib were intragastric administered at a dose of 20 mg kg^−1^ per day. Tumor size was measured every 2 d by vernier caliper. The laboratory animals were maintained under standard conditions and raised following the National Research Council's guide. The average tumor volume in each group was calculated based on the equation for a prolate spheroid (tumor volume = (short‐diameter)^2^ × large‐diameter × π/6) and expressed in mm^3^.

For UM liver metastasis mouse model, 4‐week‐old BALB/c‐nu/nu mice were injected intrasplenically with 2.5×10^6^ MUM2B‐EGFP and MUM2B‐VGF‐EGFP cells resuspended in 25 µL of PBS. The mice were randomly separated into three groups (*n* = 5), namely MUM2B‐EGFP, MUM2B‐VGF‐EGFP, and MUM2B‐VGF‐EGFP + GQ127. For MUM2B‐VGF‐EGFP + GQ127, GQ127 was intraperitoneally injected every day with a dose of 10 mg kg^−1^, and the other was replaced with the same amount of solvent. Liver metastasis was detected by ex vivo bioluminescence imaging using the IVIS Spectrum (PerkinElmer, USA).

### Bioinformatic Analysis

The GSE33655, GSE66048, GSE197656, GSE176345, GSE181125, GSE84976, GSE22138 cohorts, and corresponding platform annotation files (GPL files) were downloaded from the Gene Expression Omnibus (GEO) database (http://www.ncbi.nlm.nih.gov/geo/) and subsequently analyzed by GEO2R in GEO website or R (Version 3.4, http://www.bioconductor.org) with edgeR package. Fold‐change of gene expression in our secretome, GSE33655, GSE66048, and our RNA seq data (siGNAQ_vs_siContrl and shVGF_vs_shCtrl) were calculated with threshold criteria of Fold change [FC] > 2, *p* < 0.05. The online database of GEPIA, a website of the Genomics Analysis and Visualization Platform (http://gepia.cancer‐pku.cn/index.html) was used to determine the clinical survival of the related genes was recruited to determine the clinical survival of the related genes or signature and the correlation analysis. The group cutoff for determining clinical survival of VGF was high‐50%/low‐50% in UVM‐TCGA database. The group cutoff for determining clinical survival of TGF‐β signaling signature was high‐25%/low‐75% in UVM‐TCGA database. Kyoto Encyclopedia of Genes and Genomes (KEGG) pathway enrichment analysis was performed to investigate the activated pathways of the DEGs, by applying an online tool of DAVID Bioinformatics Resources 6.8 (http://david.ncifcrf.gov). GSEA analysis was performed using the Java desktop software (http://software.broadinstitute.org/gsea/index.jsp). Genes were ranked according to the shrunken limma log2 fold changes, and the GSEA tool was used in “Pré‐ranked” mode with all default parameters. The open‐access database of transcription factor binding profiles‐JASPAR 2020 (http://jaspar.genereg.net/) was recruited to predict related motifs.

### Statistical Analysis

Statistical analysis was performed by GraphPad Prism 8.0 software (GraphPad, Inc., La Jolla, CA, USA) and quantitative data were presented as the mean ± SD unless stated otherwise. Data were analyzed by unpaired Student's *t*‐test, one‐way ANOVA, or Pearson Chi‐square test. The sample sizes (*n*) and probability (*p*) values for each experiment were indicated in detail in figure legends. Survival curves were constructed using the Kaplan–Meier method and analyzed by the log‐rank test. The correlations analysis was assessed by Pearson's correlation analysis. To represent the results as a heat map, the quantitated minimum and maximum values were shown with the lowest and highest color intensity, respectively, and the remaining values were ranked according to their relative values. Differences between values were considered statistically significant when *p < 0.05, **p < 0.01, ***p < 0.001, or ****p < 0.0001.

### Ethics Approval

The animal procedures were approved by the Institutional Animal Care and Use Committee of Sun Yat‐sen University and followed the Guide for the Care and Use of Laboratory Animals.

## Conflict of Interest

The authors declare no conflict of interest.

## Author Contributions

S.O., S.S., and W.D. contributed equally to this work. S.O. and S.S.: Conception, formal analysis, investigation, data curation, writing–original draft. W.D. and Y.G.: Resources, investigation, methodology. Y.S. and J.M.: Data curation, investigation, methodology. K.P., Q.Z., and G.L.: Methodology, investigation. W.X., P.Y., and J.L.: Methodology, writing–review, and editing. X.Z., X.X., and Y.W.: Conceptualization, design, supervision, funding acquisition, writing–review, and editing.

## Supporting information



Supporting Information

## Data Availability

The data that support the findings of this study are available on request from the corresponding author. The data are not publicly available due to privacy or ethical restrictions.
