# Supplementary material for: Neuropeptide Precursor VGF Promotes Liver Metastatic Colonization of Gαq Mutant Uveal Melanoma by Facilitating Tumor Microenvironment via Paracrine Loops
Source: Adv Sci (Weinh). 2024 Oct 18;11(46):2407967. doi: 10.1002/advs.202407967 (PMC11633529; doi:10.1002/advs.202407967)
Supplement: Supplementary file 1 — Supporting Information [file ADVS-11-2407967-s001.docx]

**Supplementary materials for**

**Neuropeptide Precursor VGF Promotes Liver Metastatic Colonization of Gαq Mutant Uveal Melanoma by Facilitating Tumor Microenvironment via Paracrine Loops**

Shumin Ouyang^#1^, Shuo Shi^#1^, Wen Ding^#1^, Yang Ge^1^, Yingxue Su^2^, Jianshan Mo^1^, Keren Peng^1^, Qiyi Zhang^1^, Guopin Liu^2^, Wei Xiao^2^, Peibin Yue^3^, Jinjian Lu^4^, Yandong Wang^2^*, Xiaofeng Xiong^1^*, Xiaolei Zhang^1^*

1. National-Local Joint Engineering Laboratory of Druggability and New Drug Evaluation, Guangdong Key Laboratory of Chiral Molecule and Drug Discovery, School of Pharmaceutical Sciences, Sun Yat-sen University, Guangzhou, 510006, China

2. State Key Laboratory of Ophthalmology, Zhongshan Ophthalmic Center, Sun Yat-sen University, Guangzhou 510060, China

3. Department of Medicine, Division of Hematology-Oncology, and Samuel Oschin Comprehensive Cancer Institute, Cedars-Sinai Medical Center, Los Angeles, CA, 90048, USA

4. State Key Laboratory of Quality Research in Chinese Medicine, Institute of Chinese Medical Sciences, University of Macau, Macao, 999078, China

#These authors contributed equally to this work.

*To whom correspondence should be addressed:

E-mail:

zhangxlei5@mail.sysu.edu.cn (Xiaolei Zhang)

xiongxf7@mail.sysu.edu.cn (Xiaofeng Xiong)

wangydsyj@163.com (Yandong Wang)

**Supplemental Figure1-5**

**Supplemental Table1-4**

**Supplemental Figure1-****5**


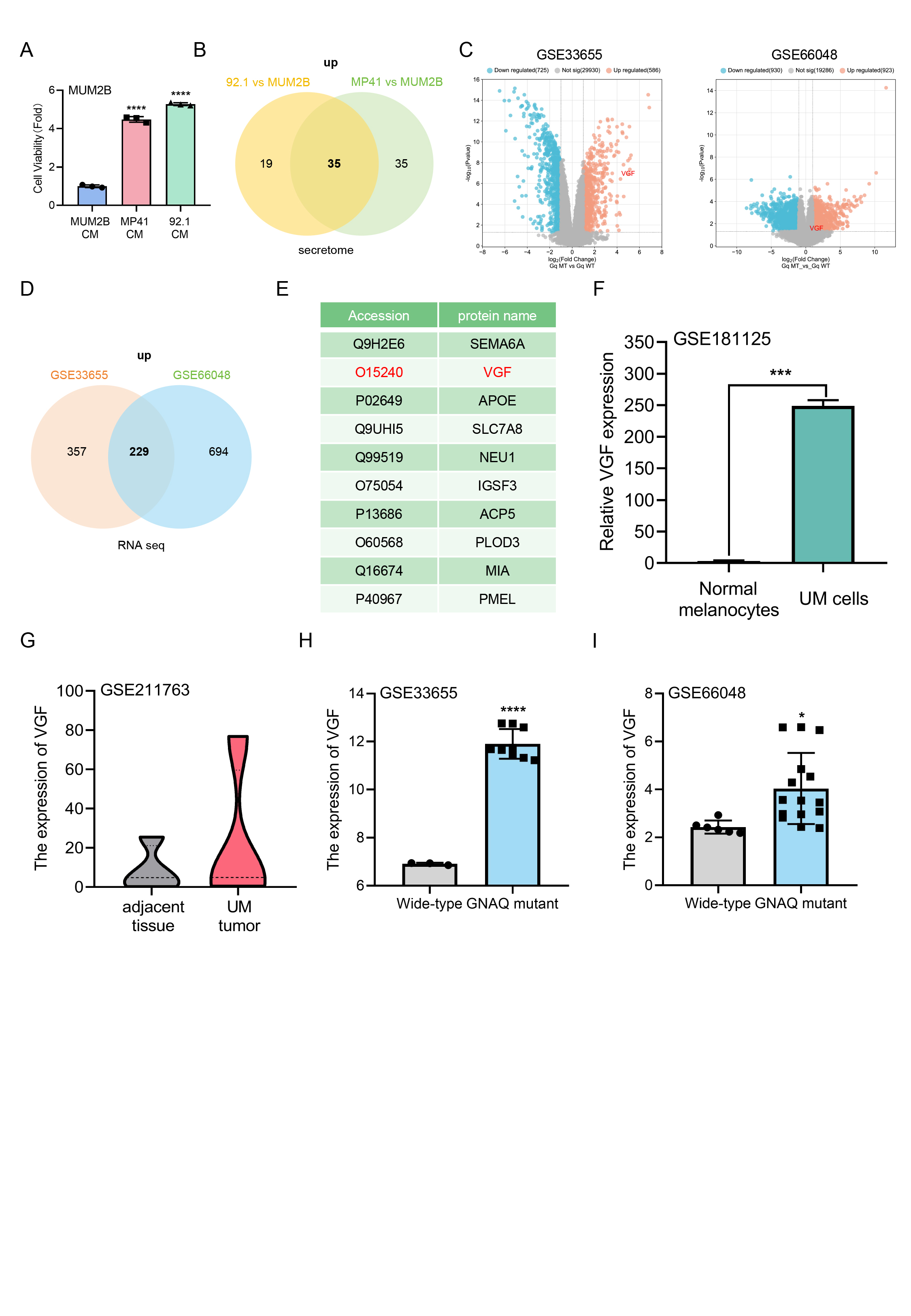


**Figure S1** **VGF secretion and expression are associated with Gαq mutant UM** **(related to Figure 1)**

**A**) Cell viability determined by CCK8 assay in MUM2B cells after cultured with MUM2B, MP41 or 92.1 CM for 72 h. Data were presented as mean ± SD, *n* = 3. ****P < 0.0001 by using the one-way ANOVA. **B**) Venn diagrams showed overlapped potential targets for Gαq mutant UM cells by secretome analysis. **C**) Volcano plot showing genome-wide mRNA expression in Gαq mutant UM cells compared with Gαq wild-type UM cells in GEO database (left: GSE33655; right: GSE66048). Fold change [FC] > 2, *p* < 0.05. **D**) Venn diagrams showed overlapped potential targets for Gαq mutant UM cells by RNA seq in the GEO database. **E**) The 10 extracellular proteins were identified as consistently upregulated in Gαq mutant UM by overlapping analysis of the secretome data and RNA seq data (GEO: GSE33655 and GSE66048). **F**) The expression of VGF in UM cells compared with normal melanocytes in GSE181125. Data were presented as mean ± SD, *n* = 2. ****p* < 0.001 by using two-tailed unpaired Student *t*-test. **G**) The expression of VGF in UM tumor compared with adjacent tissue in GSE211763. **H**) The expression of VGF in GNAQ mutant UM cells compared with GNAQ wild-type UM cells in GSE33655. Data were presented as mean ± SD, wild type group (n=3), GNAQ mutant group (n=9). ****P < 0.0001 by using two-tailed unpaired Student *t*-test. **I**) The expression of VGF in GNAQ mutant UM cells compared with GNAQ wild-type UM cells in GSE66048. Data were presented as mean ± SD, wild type group (n=6), GNAQ mutant group (n=15). *P < 0.05 by using two-tailed unpaired Student *t*-test.


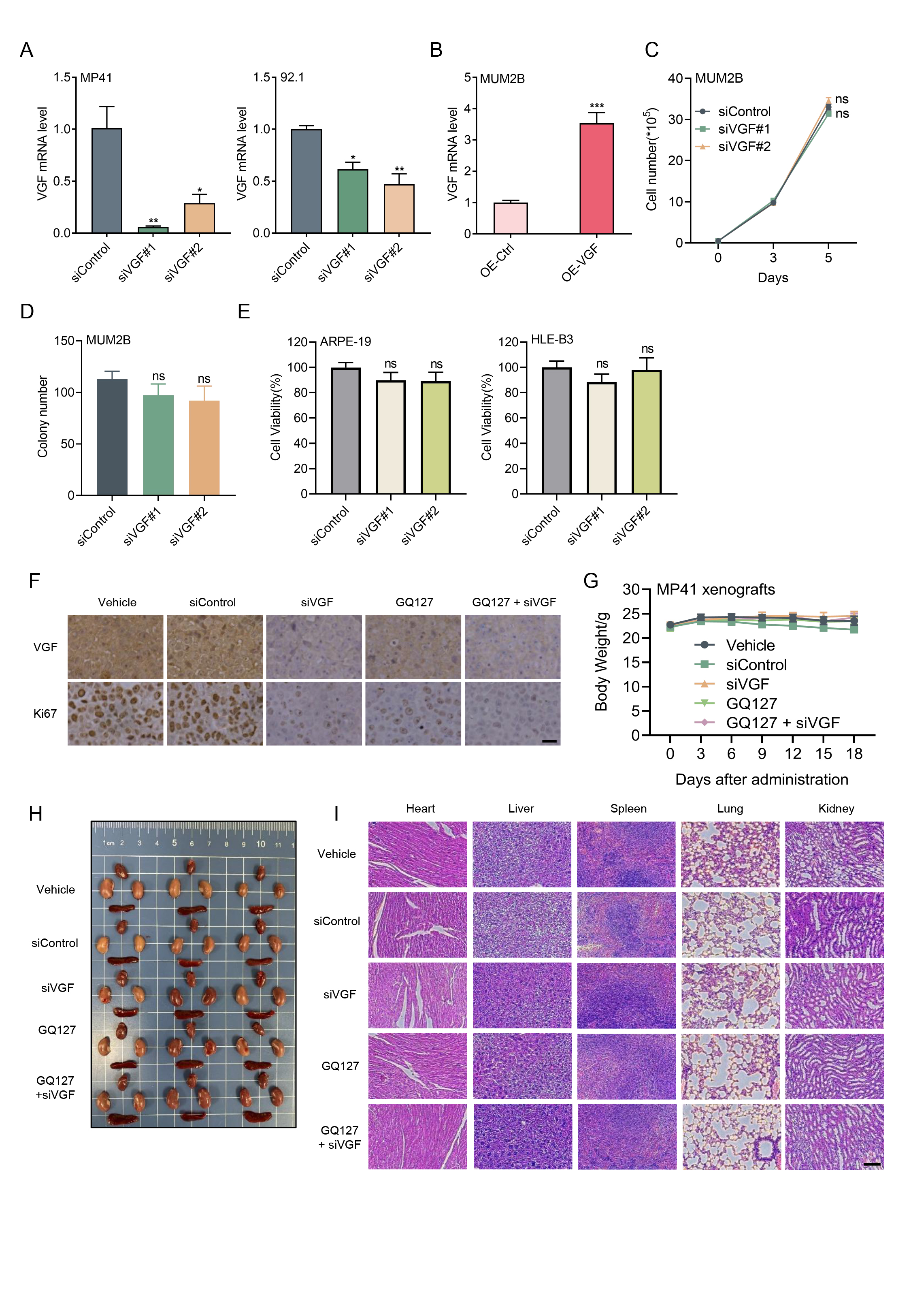


**Figure S2** **VGF promotes UM cell proliferation *in vitro* and *in vivo* (related to Figure 2)**

**A**) The knockdown effect of VGF in MP41 and 92.1 cells were determined by RT-qPCR analysis. Data were presented as mean ± SD, *n* = 2. *P < 0.05; **P < 0.01 by using the one-way ANOVA. **B**) The effect of VGF overexpression in MUM2B cells were determined by RT-qPCR analysis. Data were presented as mean ± SD, *n* = 2. ****p* < 0.001 by using two-tailed unpaired Student *t*-test. **C**) Cell growth determined by cell numbers in MUM2B cells treated with siRNAs against VGF or control siRNA. Viable cells were counted at indicated time points (day3 and day5). Data were presented as mean ± SEM, *n* = 3. ns, not significant. Ns by using the one-way ANOVA. **D**) Cell survival determined by colony formation in MUM2B cells treated with siRNAs against VGF or control siRNA. Data were presented as mean ± SD, *n* = 3. ns, not significant. Ns by using two-tailed unpaired Student *t*-test. **E**) Cell viability determined by cell numbers in non-tumor cells (ARPE-19, a human retinal pigment epithelial cell line, and HLE-B3, a human lens epithelial cell line) treated with siRNAs against VGF or control siRNA (siControl) for 3 days. Data were presented as mean ± SD, *n* = 3. ns, not significant. Ns by using two-tailed unpaired Student *t*-test. **F**) Immumohistochemical staining images of the indicated proteins in the randomly selected xenograft tumor section. Scale bars = 50 μm. **G-I**) BALB/c-nu/nu mice bearing the MP41 subcutaneous xenografts received the vehicle, GQ127 (intraperitoneal injection, 10 mg/kg), once daily; siVGF or siControl (intratumoral injection) every 3 days (n = 10 per group). The body weight of the mouse (**G**), the representative images (**H**), and HE images (**I**) of heart, liver, spleen, lung, and kidney are shown. Scale bars = 100 μm.


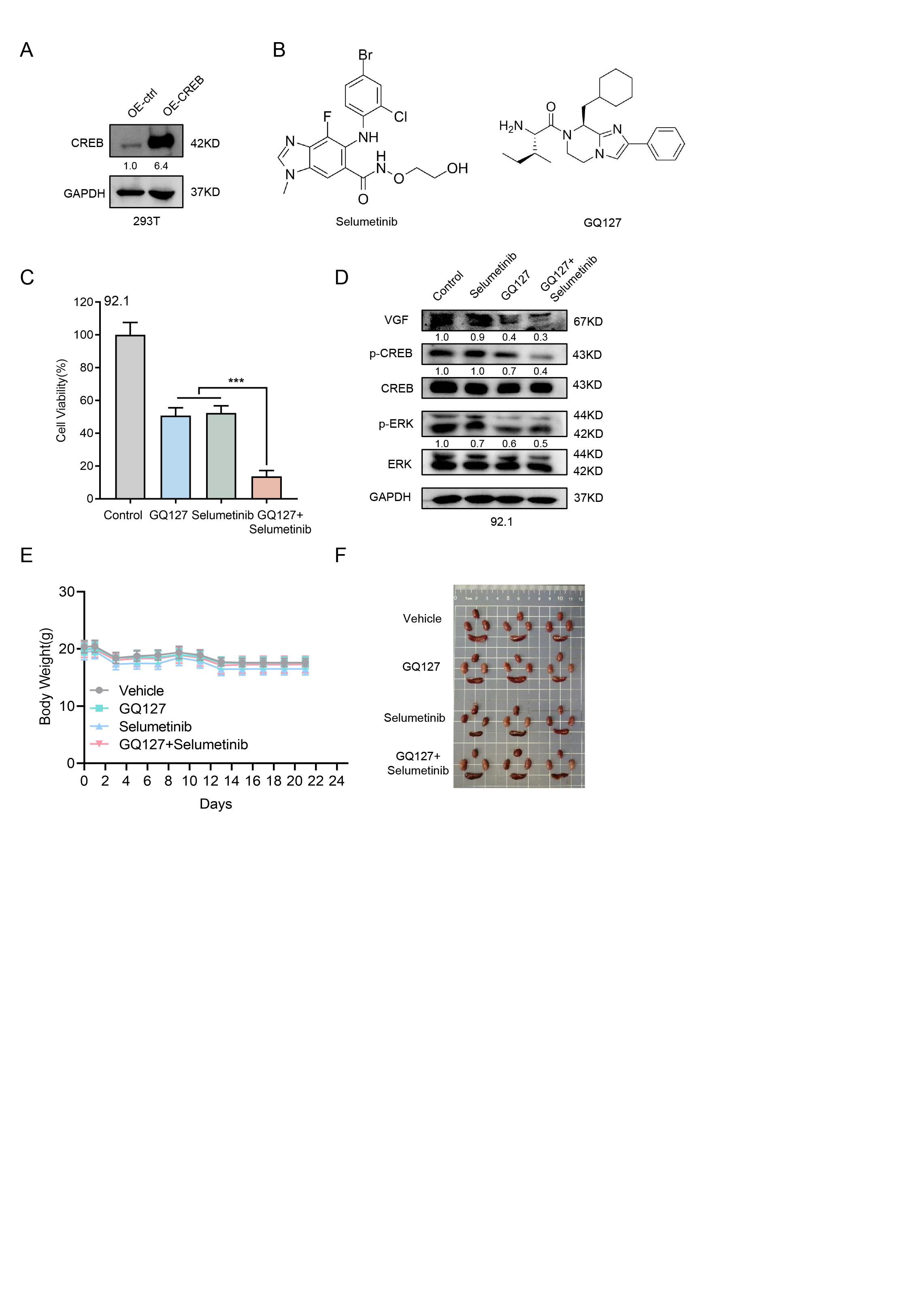


**Figure S3 VGF is regulated by Gαq through MAPK/CREB pathway in** **Gαq mutant UM (related to Figure 3)**

**A**) The overexpression of CREB level in 293T cells was determined by immunoblotting analysis. **B**) The chemical structure of Selumetinib and GQ127. **C**) Cell growth determined by cell numbers in 92.1 cells treated with GQ127(15 μM) or Selumetinib (1 μM) for 72 h. Data were presented as mean ± SD, n=3. ****p* < 0.001 by using two-tailed unpaired Student *t*-test. **D**) Immunoblotting of the indicated proteins in 92.1 cells treated with GQ127 (15 μM) or selumetinib (15 μM) for 24 h. **E-F**) Nude mice bearing MP41 xenografts received GQ127 (10 mg/kg, i.p, daily), Selumetinib (20 mg/kg, oral, daily), or vehicle daily (n=6 per group). The body weight of the mouse (**E**) and the representative images (**F**) of the heart, spleen, and kidney.


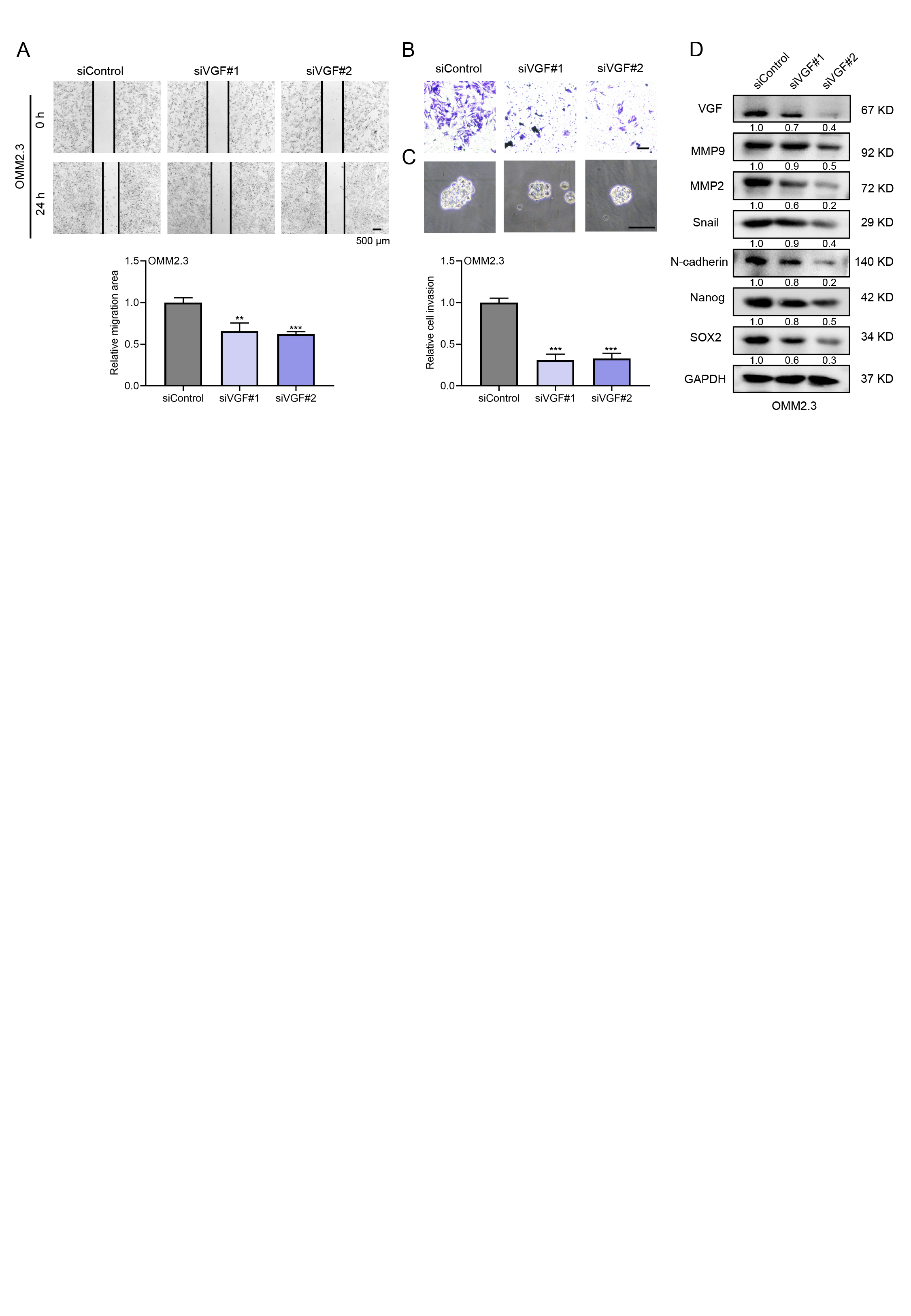


**Figure S4 VGF promotes migration and invasion of Gαq mutant uveal melanoma cells (related to Figure 4)**

**A**) Cell migration determined by wound healing assay in OMM2.3 cells treated with siRNAs against VGF or control siRNA. Scale bars = 500 μm. Bottom: quantification of cell migration (Fold change). Data were presented as mean ± SD, n=3. **P < 0.01, ****p* < 0.001 by using two-tailed unpaired Student *t*-test. **B**) Cell invasion determined by transwell assay in OMM2.3 cells treated with siRNAs against VGF or control siRNA. Scale bars = 200 μm. Bottom: quantification of cell invasion (Fold change). Data were presented as mean ± SD, n=3. ****p* < 0.001 by using two-tailed unpaired Student *t*-test. **C**) Cell stemness determined by spheroid formation assay in OMM2.3 cells treated with siRNAs against VGF or control siRNA for 7 days. Scale bars = 200 μm. **D**) Immunoblotting of the indicated proteins in OMM2.3 cells treated with siControl and siVGF (#1 and #2) for 72 h.

**
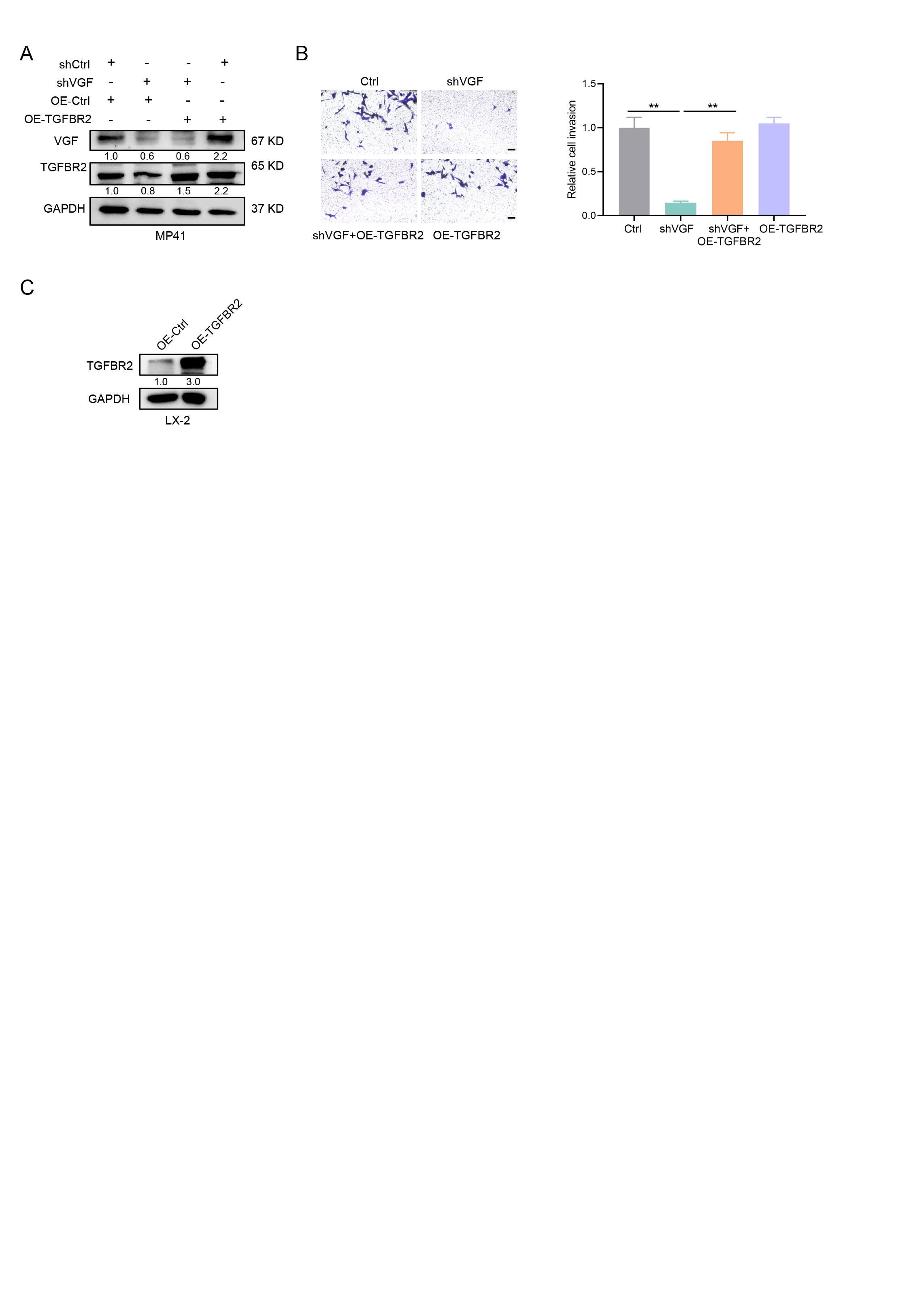
**

**Figure S5 VGF promotes uveal melanoma growth and metastasis by regulating TGF-β-SMAD signaling pathway** **(related to Figure 6 and Figure 7)**

**A**) Immunoblotting of the indicated proteins in MP41 (shCtrl, shVGF and shVGF &OE-TGFBR2) cell lines. **B**) Cell invasion detected by transwell assay in MP41 (shCtrl, shVGF and shVGF&OE-TGFBR2) cell lines. Scale bars = 200 μm. Right: quantification of cell invasion (Fold change). Data were presented as mean ± SD, n=3. **P < 0.01 by using two-tailed unpaired Student *t*-test. **C**) The overexpression of TGFBR2 level in LX-2 cells was determined by immunoblotting analysis.

**Supplemental Table1-4**

**Table S1 The primer sequences for siRNA and shRNA used in this study**

| Construct | Species | Direction | Sequence (5' - 3') |
| --- | --- | --- | --- |
| siVGF#1 | Human |  | GAAUUACAUCGAGCACGUGCU |
| siVGF#2 | Human |  | GACGAUCGACAGCCUCAUUGA |
| siGNAQ#1 | Human |  | CUAUGAUAGACGACGAGAAUA |
| siGNAQ#2 | Human |  | CCUCGGUUAUUCUGUUCUUAA |
| siGNAQ#3 | Human |  | GCACAAUUAGUUCGAGAAGUU |
| shVGF | Human | Forward | CCGGCTCAGCTCTGAGCATAAAGAGCTCGAGCTCTTTATGCTCAGAGCTGAGTTTTTG |
|  | Human | Reverse | AATTCAAAAACTCAGCTCTGAGCATAAAGAGCTCGAGCTCTTTATGCTCAGAGCTGAG |

**Table S2 The primer sequences for RT-qPCRs used in this study**

| Construct | Species | Direction | Sequence (5' - 3') |
| --- | --- | --- | --- |
| GAPDH | Human | Forward | GAGAAGGCTGGGGCTCATTT |
|  | Human | Reverse | AGTGATGGCATGGACTGTGG |
| GNAQ | Human | Forward | TCAACGACGAGATCGAGCGG |
|  | Human | Reverse | AGCTTGGTGAAGCCCCTTTTA |
| VGF | Human | Forward | GGAACTGCGAGATTTCAGTCC |
|  | Human | Reverse | GTGCGGGTTTCCGTCTCTG |
| SNAI1 | Human | Forward | TGCCCTCAAGATGCACATCCGA |
|  | Human | Reverse | GGGACAGGAGAAGGGCTTCTC |
| SNAI2 | Human | Forward | ATCTGCGGCAAGGCGTTTTCCA |
|  | Human | Reverse | GAGCCCTCAGATTTGACCTGTC |
| CDH2 | Human | Forward | CCTCCAGAGTTTACTGCCATGAC |
|  | Human | Reverse | GTAGGATCTCCGCCACTGATTC |
| VIM | Human | Forward | AGGCAAAGCAGGAGTCCACTGA |
|  | Human | Reverse | ATCTGGCGTTCCAGGGACTCAT |
| VEGFA | Human | Forward | TTGCCTTGCTGCTCTACCTCCA |
|  | Human | Reverse | GATGGCAGTAGCTGCGCTGATA |
| CLDN6 | Human | Forward | GTGGAAGGTGACCGCTTTCATC |
|  | Human | Reverse | CAGCAGTGAGTCGTACACCTTG |
| MMP2 | Human | Forward | GCTGCATCCAGACTTCCTCAG |
|  | Human | Reverse | TCCATCGTAGCGCTCCCT |
| MMP9 | Human | Forward | TCTATGGTCCTCGCCCTGAA |
|  | Human | Reverse | CATCGTCCACCGGACTCAAA |
| Nanog | Human | Forward | CTCCAACATCCTGAACCTCAGC |
|  | Human | Reverse | CGTCACACCATTGCTATTCTTCG |
| SOX2 | Human | Forward | GCTACAGCATGATGCAGGACCA |
|  | Human | Reverse | TCTGCGAGCTGGTCATGGAGTT |
| EP300 | Human | Forward | GATGACCCTTCCCAGCCTCAAA |
|  | Human | Reverse | GCCAGATGATCTCATGGTGAAGG |
| ID1 | Human | Forward | GTTGGAGCTGAACTCGGAATCC |
|  | Human | Reverse | ACACAAGATGCGATCGTCCGCA |
| ID3 | Human | Forward | CAGCTTAGCCAGGTGGAAATCC |
|  | Human | Reverse | GTCGTTGGAGATGACAAGTTCCG |
| ID4 | Human | Forward | GGACCTGTCCAGCCGCGCC |
|  | Human | Reverse | TCAGCGGCACAGAATGCTGTCG |
| SMAD1 | Human | Forward | TTGGCACAGTCTGTGAACCATGG |
|  | Human | Reverse | GTAACATCCTGGCGGTGGTATTC |
| SMAD2 | Human | Forward | GGGTTTTGAAGCCGTCTATCAGC |
|  | Human | Reverse | CCAACCACTGTAGAGGTCCATTC |
| SMAD3 | Human | Forward | TGAGGCTGTCTACCAGTTGACC |
|  | Human | Reverse | GTGAGGACCTTGTCAAGCCACT |
| SMAD6 | Human | Forward | CACTGAAACGGAGGCTACCAAC |
|  | Human | Reverse | CCTGGTCGTACACCGCATAGAG |
| SMAD9 | Human | Forward | GTGCTGTGAGTTCCCATTTGGC |
|  | Human | Reverse | TTCACTGTGTCTTGGCACGAGC |
| TGFBR2 | Human | Forward | GTCTGTGGATGACCTGGCTAAC |
|  | Human | Reverse | GACATCGGTCTGCTTGAAGGAC |
| BMP6 | Human | Forward | CCGACAACAGAGTCGTAATCGC |
|  | Human | Reverse | CTGCCATCCCAGGTCTTGGAAA |
| BMPR1B | Human | Forward | CTGTGGTCACTTCTGGTTGCCT |
|  | Human | Reverse | TCAATGGAGGCAGTGTAGGGTG |
| BMPR2 | Human | Forward | AGAGACCCAAGTTCCCAGAAGC |
|  | Human | Reverse | CCTTTCCTCAGCACACTGTGCA |
| CTGF | Human | Forward | CTTGCGAAGCTGACCTGGAAGA |
|  | Human | Reverse | CCGTCGGTACATACTCCACAGA |
| ACTA2 | Human | Forward | CTATGCCTCTGGACGCACAACT |
|  | Human | Reverse | CAGATCCAGACGCATGATGGCA |
| COL1A1 | Human | Forward | GATTCCCTGGACCTAAAGGTGC |
|  | Human | Reverse | AGCCTCTCCATCTTTGCCAGCA |
| FN1 | Human | Forward | ACAACACCGAGGTGACTGAGAC |
|  | Human | Reverse | GGACACAACGATGCTTCCTGAG |
| TGFB1 | Human | Forward | TACCTGAACCCGTGTTGCTCTC |
|  | Human | Reverse | GTTGCTGAGGTATCGCCAGGAA |

**Table S3 The primer sequences for Chip-qPCR used in this study**

| Construct | Species | Direction | Sequence (5' - 3') |
| --- | --- | --- | --- |
| CREB1 | Human | Forward | CTAGCTCGCTCCGGCTTC |
|  | Human | Reverse | CAGGCACCCTCCAATCGTC |

**Table S4 The primer sequences for reporter-gene assays used in this study**

| Construct | Species | Direction | Sequence (5' - 3') |
| --- | --- | --- | --- |
| VGF-WT | Human | Forward | CCCTCGAGAGGGTTTGAGGGACGAACAGC |
|  | Human | Reverse | CCAAGCTTTTCTATTCCACTCAGCAACGCG |
| VGF-Mut | Human | Forward | TATTTACCAGCTGGTGGTCCGACGCGAGAG |
|  | Human | Reverse | GACCACCAGCTGGTAAATACTCCGCTGTTCGT |
